# Heterotrophic Prokaryote Host–Virus Dynamics During Spring in the Northeast Atlantic Ocean

**DOI:** 10.3390/microorganisms13112474

**Published:** 2025-10-29

**Authors:** Yean Das, Corina P. D. Brussaard, Kristina D. A. Mojica

**Affiliations:** 1 Division of Marine Science, School of Ocean Science and Engineering, The University of Southern Mississippi, Stennis Space Center, Hancock County, MS 39529, USA; yean.das@usm.edu; 2 Department of Marine Microbiology and Biogeochemistry, NIOZ—Royal Netherlands Institute for Sea Research, 1790 AB Den Burg, The Netherlands; 3 Department of Freshwater and Marine Ecology, Institute for Biodiversity and Ecosystem Dynamics (IBED), University of Amsterdam, 1000 GG Amsterdam, The Netherlands

**Keywords:** heterotrophic prokaryotes, bacterial production, viral lysis, HNA, LNA, marine viruses, viral production, North Atlantic Ocean, spring, lysogeny

## Abstract

Flow cytometry typically reveals two heterotrophic prokaryote (HP) subpopulations when stained with SYBR Green: high nucleic acid (HNA) and low nucleic acid (LNA) cells. Evidence suggests these populations have distinct physiological and ecological roles with implications for mortality. We assessed HP abundance, production, the relative proportion of HNA and LNA, virus-mediated mortality, and the distribution of lytic versus lysogenic strategies within HP host communities across a latitudinal gradient in the North Atlantic during spring. The study area, characterized by dynamic physicochemical conditions consistent with the onset of seasonal stratification, was divided into three regions based on bio-physicochemical properties: Pre-bloom, Bloom, and Oligotrophic. Multivariant analysis showed these regions significantly structured HPs, as well as influenced the relative abundance and production of virus subpopulations (i.e., V1 and V2). Specifically, V1 viruses increased with the potential of encountering HNA hosts, which were elevated in the surface waters of stratified Oligotrophic and Bloom regions. In contrast, V2 abundance and production correlated with LNA cells, more prominent in DEEP samples and in surface waters of the deeper mixed Pre-bloom region. Lysogeny occurred across all regions, with the percentage of lysogens within the HP community, increasing (largely V1-driven) with HP-specific growth rate until reaching a threshold of 0.1 d^−1^, after which it declined. We discuss the potential ecological underpinnings driving these patterns and implications for carbon flux.

## 1. Introduction

Bacteria and Archaea are two major evolutionary domains of life and together represent the largest fraction of living biomass in the ocean [1,2]. Within these domains, heterotrophic prokaryotes (HPs) play a vital role in global biogeochemical cycles through the remineralization of dissolved organic matter (DOM) and subsequent synthesis into new biomass. Flow cytometry is commonly used to enumerate and characterize HPs in aquatic systems; however, it does not permit the distinction between heterotrophic bacteria and archaea. Accordingly, they are collectively referred to here as HPs. When stained with SYBR Green, flow cytometric analysis typically reveals two distinct subpopulations that display similar side scatter but differ in their green fluorescence intensity (Figure S1), which reflects variation in cellular DNA content [3,4]. These populations are commonly referred to as high nucleic acid (HNA) and low nucleic acid (LNA) cells. Although both subpopulations are prevalent in aquatic systems [5,6], their relative distributions can vary across environmental conditions and geographic regions [7,8,9]. Numerous studies have focused on examining variations in the composition and activity of these two subpopulations across physicochemical gradients [6,10,11]. These studies have found that HNA cells are typically associated with productive environments, whereas LNA cells are generally more abundant in oligotrophic regions, suggesting physiological and ecological differences between these two populations [9] and potential for distinct roles in organic matter cycling in the ocean. However, little is still known about the processes that shape their relative distributions over broad geographical scales.

Mortality processes play a significant role in shaping the biomass, composition, and activity of marine microbial communities [12,13]. Among these processes, viruses have been recognized over the past few decades as a major mortality agent of marine HPs [14,15,16]. Viruses are the most abundant biological entities in the ocean, with average concentrations of ~10^8^ mL^−1^ in surface waters [17,18,19,20], and the majority of these are bacteriophages [9,21]. Viruses exhibit diverse infection strategies with lytic and lysogenic infection modes being the most prevalent [18,19,22,23,24]. Lytic infection (resulting in host cell lysis) exhibits density dependence, and under the “Kill-the-winner” paradigm, viruses are believed to target the most abundant and metabolically active microbial populations within natural assemblages. Lysogeny (virus integrates its genetic material into the host cell’s genome) is typically regarded as a survival strategy that allows viruses to persist under conditions of low host-density and productivity, yet it has been observed across both low and high host densities [25]. Collectively, these findings underscore the sensitivity of host–virus interactions to both biological and environmental conditions, suggesting that physical processes influencing host distributions and activity have the potential to fundamentally alter viral infection dynamics.

Several studies provide evidence that physiological and ecological differences between HP subpopulations, LNA and HNA cells, can alter viral production rates [9,14,24]. Similar to their hosts, flow cytometric analysis of viruses stained with SYBR Green reveals distinct subpopulations that share similar side scatter (SSC, related to cell size) but differ in green fluorescence intensity. These groups are commonly referred to as V1, V2, and V3, as the fluorescence signal is not related to nucleic acid content in viruses (Figure S1). Recently, Mojica et al. (2020) reported group- and activity-specific linkages between certain viral subpopulations and HP host subpopulations (HNA-V1 and LNA-V2), with implications for the magnitude of carbon flux mediated by viral infection [9]. These virus–host pairings were also found to vary with changes in the surrounding physical eddy field, suggesting a potential role of hydrodynamic processes in structuring these interactions.

Despite these advances, the influence of physical forcing on virus–host interactions remains poorly resolved. Variability in the abundance and activity of specific virus–HP pairings may help explain the recently observed inverse relationship between turbulence (measured as temperature-driven eddy diffusivity) and rates of lytic viral production during summer [14]. However, summer represents a period of strong, stable stratification, when turbulence is relatively low and microbial communities are already well established. In contrast, spring marks the onset of stratification, a transitional period characterized by rapidly shifting physical conditions and strong mixing events that can alter the distribution and encounter rates of host and viral populations. Investigating host–virus dynamics during this window provides a unique opportunity to determine how emerging stratification influences microbial interactions and viral control of carbon flux. Specifically, to examine the overarching hypothesis that host–virus interactions are governed not only by host availability and physiology, but also by physical drivers that structure microbial communities and regulate carbon cycling across large spatial scales.

In this study, we revisited the same transect in the North Atlantic as Mojica & Brussaard, 2020 [14], to examine how emerging stratification influences host–virus dynamics. We assessed heterotrophic prokaryote abundance and production, the relative proportion of LNA and HNA cells in the HP community, viral-mediated mortality, and the relative distribution of virus reproductive strategies (lytic versus lysogenic) within HP host communities over a latitudinal gradient across the North Atlantic in the spring, when the water column was transitioning from well-mixed (North) to weakly stratified (South).

More explicitly, we evaluated two sets of hypotheses: (H1) The relative abundance of HP subpopulations (HNA and LNA) and the total HP production vary according to phytoplankton biomass (Phyto C) reflecting differences in carbon availability in the water column, and (H1b) these relationships are strongly influenced by the water column stratification. (H2) The relative abundance of virus subpopulations (specifically V1 and V2) and their lytic production rates vary according to the relative abundance and production of HP host subpopulations (HNA and LNA). In this framework, the more productive HNA cells are expected to be associated with the more abundant and productive V1 virus group, whereas LNA cells would correspondingly be linked to V2.

## 2. Materials and Methods

### 2.1. Study Area and Physicochemical Parameters

**Figure 1:**
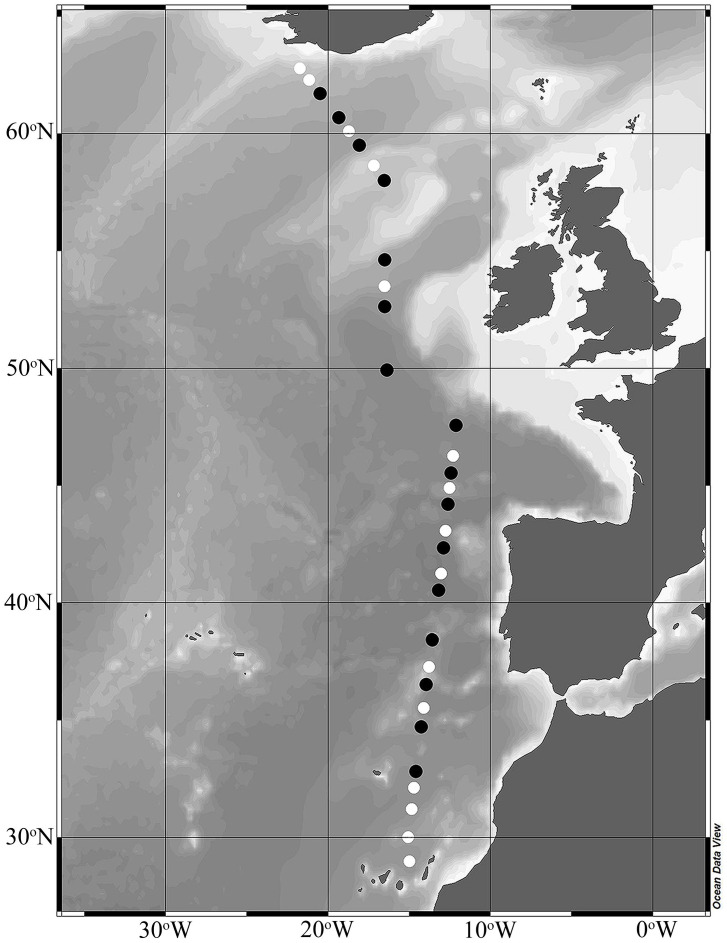
Bathymetric map of stations sampled during the spring (April–May) STRATIPHYT II cruise. Incubation experiments for heterotrophic prokaryote production and viral-mediated mortality were conducted at stations indicated by black symbols. The figure was prepared using Ocean Data View (version 5.5.1).

In the spring (April–May) of 2011, 32 stations (each separated by approximately 100 km) were sampled along a North–South transect in the Northeast Atlantic Ocean during the STRATIPHYT II cruise, which took place onboard the R/V Pelagia (Figure 1). Water samples were collected prior to dawn at each station from at least 10 separate depths using 24 plastic samplers (General Oceanics type Go-Flow, 10 L) mounted on a trace-metal clean system framed with a fully titanium sampler equipped with a CTD (Seabird 9+; standard conductivity, temperature, and pressure sensors) system and auxiliary sensor for chlorophyll autofluorescence (Chelsea Aquatracka Mk III). Chlorophyll autofluorescence sensor data were calibrated against HPLC data according to van de Poll et al. [26]. All water samples were collected inside a trace-metal clean container and handled according to Eich et al. [27]. Phytoplankton carbon biomass (PhytoC) was determined by applying size-specific carbon conversion factors to phytoplankton cell abundances (PhytoA) obtained using flow cytometry [28,29,30].

Processing of physicochemical variables and data have been discussed previously [30,31,32]. Temperature eddy diffusivity (K_T_), referred here as vertical mixing coefficient, was determined from temperature and conductivity microstructure profiles measured from SCAMP (Self Contained Autonomous Microprofiler) deployed at 17 stations and down to 100 m depth [32,33]. For additional stations and depths, data were interpolated using the spatial kriging function ‘krig’ executed in R (version R 3.0.1) using the ‘fields’ package [34]. Water column stratification was quantified using Brunt–Väisälä Buoyancy frequency (N^2^) determined from SCAMP measurement (17 stations) or CTD data (15 stations) processed using SBE Seabird software, Version 7.23.2, (Seabird Electronics, Bellevue, WA, USA) according to Fofonoff adiabatic leveling method [35]. The stability of the water column of each station was characterized using 100 m depth-averaged N^2^ ( $\bar{N^{2}}$ ) Brunt–Väisälä Buoyancy frequency [30] and classified according to established stratification criteria as non-stratified ( $\bar{N^{2}}$ < 2 × 10^−5^ rad^2^ s^−2^), weakly stratified (2 × 10^−5^ < $\bar{N^{2}}$ < 5 × 10^−5^ rad^2^ s^2^) and strongly stratified ( $\bar{N^{2}}$ > 5 × 10^−5^ rad^2^ s^−2^). Mixed layer depth (MLD) was defined as the depth at which potential density difference with respect to the surface was 0.03 kg m^−3^ [36].

Discrete water samples for dissolved inorganic phosphate (PO_4_), ammonium (NH_4_), nitrite (NO_2_), and nitrate (NO_3_) were gently filtered through 0.2 µm pore size polysulfone Acrodisk filters (32 mm, Pall Corp., Port Washington, NY, USA) and stored at –20 °C until analysis. Dissolved inorganic nutrients were analyzed onboard with a Bran + Luebbe Quaatro Auto-Analyzer for dissolved orthophosphate [37], ammonium [38,39] and inorganic nitrogen (nitrate + nitrite: NO_x_) [40] with the following detection limits: 0.01 µM for PO_4_, 0.05 µM for NH_4_, and 0.06 µM for NO_x_.

Water samples for rate-based measurements (i.e., heterotrophic prokaryote production and viral production) were obtained from 2–3 separate depths at 16 stations along the cruise transect (Figure 1, black symbols) and categorized as SURFACE euphotic zone (0–90 m), which included the deep chlorophyll maximum (DCM) when present, as defined by the presence of a subsurface peak in the vertical profile of Chl *a* autofluorescence [26,30] and DEEP samples (90–200 m).

### 2.2. Microbial Abundances

Heterotrophic prokaryotes (bacteria and archaea) and viruses were enumerated using a Becton-Dickinson FACSCalibur flow cytometer (FCM) stocked with an air-cooled Argon laser with an excitation wavelength of 488 nm (15 mW) according to Marie et al. (1999) and Brussaard (2004), with modifications by Mojica et al. (2014) [41,42,43]. In short, samples were fixed with 25% glutaraldehyde (EM-grade, Sigma-Aldrich, Zwijndrecht, The Netherlands) at a final concentration of 0.5% for 15–30 min at 4 °C, flash frozen in liquid nitrogen, and stored at –80 °C until analyzed. Thawed samples were diluted in TE buffer (pH 8.2, 10 mM Tris-HCL, 1 mM EDTA; Sigma-Aldrich, St. Louis, MO, USA) and stained with green fluorescent, nucleic acid-specific dye SYBR Green I (Sigma-Aldrich) and incubated in the dark for 15 at room temperature and 10 min at 80 °C for HP and virus enumeration, respectively. The trigger for analysis was set for green fluorescence and obtained. fcs format files were analyzed using the software package FlowJo (v 10.8.1). Virus (V1,V2, V3; [9]) and HP (HNA, LNA; [9]) subpopulations were distinguished based on their green fluorescence (530 nm) and 488 nm right-angle light scattered (SSC) (Figure S1). Potential contribution of photosynthetic cyanobacteria to the HNA subpopulation counts were assessed based on additional bivariate plots of green fluorescence versus red Chl *a* autofluorescence against SSC. Counts reflecting both green and red fluorescence within the HNA group were removed.

### 2.3. Heterotrophic Prokaryotic Production

Water samples for HP production (HPP) were collected directly from Niskin bottles into acid-cleaned (10% HCl and MilliQ rinsed) 10 mL polycarbonate (PC) vials. HPP was then determined according to the ^3^H-leucine incorporation method of Simon & Azam [44]. In short, quadruplicate samples were taken and one sample was used as blank to which 0.5 mL formaldehyde (37%; Sigma-Aldrich, Zwijndrecht, The Netherlands) was added to kill heterotrophs. The remaining triplicate samples were each spiked with 30 µL [^3^H] leucine (specific activity, 142.2 Ci mmol^−1^; Amersham, Little Chalfont, UK), equivalent to 50 µCi per sample, and incubated for 30–120 min in the dark at in situ temperature. Samples were then fixed with 0.5 mL formaldehyde (37%; Sigma-Aldrich, Zwijndrecht, the Netherlands) and filtered onto a 0.2 µM PC filters (25 mm, Whatman, Maidstone, UK). The filters were rinsed twice by 5% chilled trichloroacetic acid (TCA) and left to stand for 5 min. After rinsing, filters were placed into scintillation vials containing 8 mL of UltimaGold scintillation cocktail (PerkinElmer, Waltham, MA, USA) and left for 24 h. Samples were then analyzed using PerkinElmer Tricarb 2910 TR scintillation counter. Leucine incorporation rate (nmole L^−1^ h^−1^) was converted to HPP (µg L^−1^ d^−1^) assuming a cellular carbon-to-protein ratio of 0.86 and isotopic dilution factor of 2 [44,45]. Cell-specific growth rate (µ d^−1^) was determined by the following equation [46].

(1) µ=PtBt

where P_t_ = HP production rate (i.e., HPP), and B_t_ = heterotrophic biomass.

This simple method allows us to calculate µ with lower uncertainty derived from propagation of error [46]. HP biomass (Bt) was determined assuming a carbon conversion factor of 12.4 fg C cell^−1^ [47].

### 2.4. Viral-Mediated Mortality

For viral production, the virus reduction approach [48] was used. Briefly, 600 mL of seawater was concentrated to approximately 100 mL by recirculating over a 0.22 μm pore-size polyether sulfone membrane (PES) tangential flow filter (Vivaflow 50; Sartorius stedim biotech, Göttingen, Germany) at a filtrate discharge rate of 40 mL min^−1^ under in situ temperature and dimmed light conditions. After concentration, 500 mL of virus-free water (generated by 30 kDa ultrafiltration system; Vivaflow 200, PES membrane; Sartorius stedim biotech, Göttingen, Germany) was then added to the concentrated sample. This reduction–resuspension process was repeated a total of 3 times, and results in the reduction of viral concentration relative to in situ. On the final volume reduction, the volume was reduced to approximately 50 mL, and the filter was slowly backflushed to retrieve the remaining 50 mL from the system. The concentrated sample was then re-established by a final addition of 500 mL virus-free water and aliquoted into six 50 mL polycarbonate Greiner tubes (Greiner Bio-One North America Inc., Monroe, NC, USA). Viral production rates resulting from lytic infection (VP) were determined from one set of triplicate samples, while induced viral production (VPI), representing the activation of integrated (formerly lysogenic) viruses, was determined from a second set of triplicate samples following the addition of Mitomycin C (Sigma-Aldrich; final concentration 1 µg mL^−1^). Additionally, triplicate 50 mL samples of untreated whole seawater and seawater filtered through 0.2 μm pore-sized PC filters were taken to provide estimates of net HP production and viral loss rates (adsorption to cell wall), respectively. A 1 mL subsample was taken from each tube (T0) to determine virus and prokaryote abundance, and sample tubes were then incubated in the dark at in situ temperature and subsampled every 3 h for 12–24 h. Samples for abundance measurements were fixed, stored, and enumerated as described previously (see Section 2.2).

The production rate of new viruses released from infected HP cells was determined from the slope of a first-order linear regression of the change in viral concentration over time [49]. VPI (referred to as prophage induction in bacterial hosts) was calculated as the difference between the rates of VP and VPI samples. Rates were corrected for any HP loss which occurred during sample preparation and viral loss due to adsorption [48]. Virus-mediated mortality of prokaryotes (cells L^−1^ d^−1^) was estimated by dividing lytic VP rate by a burst size (BS) of 20 [50].

### 2.5. Statistical Analysis

Prior to multivariate analysis, a one-way ANOVA was applied to physicochemical measurements (e.g., temperature, salinity, density and PO_4_) to confirm that no significant differences (α < 0.05) existed among pre-dawn CTD casts. As none were detected, data were averaged across casts for use in the subsequent analysis.

Multivariate statistics was applied to the data to evaluate the hypotheses set forth in the introduction. Statistical analysis was conducted using the R statistical software (v. 2023.12) [51] supplemented with vegan [52]. Data exploration was carried out according to Zuur et al. [53]. For H1, the response variables were HNA and LNA abundance, the LNA to HNA abundance ratio (LNA:HNA), total heterotrophic prokaryote abundance (HPA; HNA + LNA), HPP, and HP-specific growth rate (µ). Explanatory variables were temperature, salinity, density, K_T_, $\bar{N^{2}}$ , oxygen (O_2_), PO_4_, NH_4_, NO_2_, NO_3_, PhytoA and PhytoC. Additionally, depth layer (2 levels: SURFACE, 0–90 m and DEEP, 90–200 m), water column stratification (2 levels: non-stratified, 0 and weakly stratified, 1) and region (3 levels: Oligotrophic (29–40 °N), Bloom (41–46 °N) and Pre-bloom (48–63 °N) were included as factors. Density and PhytoA were log-transformed, NH_4_ and PhytoC were log (x + 1)-transformed, and ( $\bar{N^{2}}$ ) was log (x + 10)-transformed to improve the homogeneity of variance and reduce the effect of outliers. Similarly, HPA and HPP from the response variables group were log- and log (x + 1)-transformed, respectively. To reduce collinearity between explanatory variables, variance inflation factors (VIFs) were calculated using the vif function in the usdm package (v. 2.1-7) [54] and explanatory variables with the largest VIF were sequentially removed until all variables resulted in VIF < 10 [55]. Any residual collinearity among explanatory variables was identified and removed based on boxplots and correlation pair plots across factor levels (i.e., depth, region, and stratification level). At this stage, depth layer was excluded due to collinearity with K_T_, PhytoA, and PhytoC. In addition, HNA and LNA were removed from the response variables group due to their high correlation with HPA (Pearson’s correlation: N = 40, *p*-value < 2.2 × 10^−16^, r = 0.99 and 0.97, respectively). This resulted in a final selection of 9 explanatory variables for H1: K_T_, $\bar{N^{2}}$ , O_2_, NH_4_, NO_2_, PhytoA, PhytoC, stratification level, and region.

Response variables for H2 were V1 virus abundance (V1), V2 virus abundance (V2), total virus abundance (VA; V1–V3), the V1 and V2 abundance ratio (V1:V2), V1 lytic production rate (VP_V1_), and V2 lytic production rate (VP_V2_). Explanatory variables were $\bar{N^{2}}$ , NO_2_, NO_3_, HPA, LNA:HNA, HPP, cell-specific prokaryotic production (HPP cell^−1^), µ, and subpopulation-specific virus to prokaryote ratio (VPR*), expressed as [(*V*2:*V*1) × *VA*]/[(*LNA*:*HNA*) × *HPA*], with stratification level and region included as factors. HPA and HPP were log- and log (x + 1)-transformed, respectively, to reduce the effect of outliers. Additionally, HPP cell^−1^ was removed from the explanatory variables due to strong collinearity with µ (Pearson’s correlation: N = 26, r = 1, *p*-value < 2.2 × 10^−16^). Similarly, V1 and V2 were removed from the response variables due to high collinearity with VA (Pearson’s correlation: N = 26, r = 0.99, *p*-value < 2.2 × 10^−16^ and r = 0.87, *p*-value = 5.0 × 10^−9^, respectively). For response variables, VA and VP_V1_ were log-transformed, and *V*1:*V*2 and VP_V2_ were log (x + 1)-transformed. Negative lytic production rates of VP_V2_ were excluded from the response variable group. After VIF analysis, 9 explanatory variables remained for H2: $\bar{N^{2}}$ , NO_2_, NO_3_, HPA, LNA:HNA, µ, VPR*, stratification level, and region.

Initial scatter plots of response variables and covariates for each hypotheses exhibited strong linear patterns and thus, redundancy analysis (RDA) was chosen over canonical correspondence analysis (CCA) [56]. Using forward selection approach, significance of explanatory variables was assessed by a permutation test with multivariate pseudo-F as test statistics [57], and only significant variables (α = 0.1) were retained in the final model. A total of 9999 permutations were calculated to estimate the *p*-values (α = 0.1) associated with the pseudo-F statistics.

## 3. Results

### 3.1. Study Area and Physicochemical Parameters

**Figure 2:**
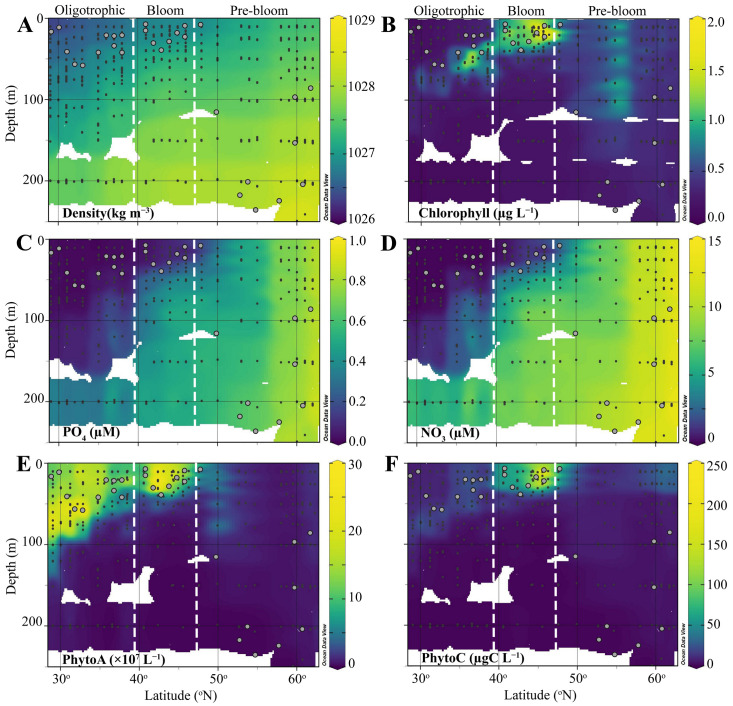
Vertical profiles of (**A**) density, (**B**) HPLC-calibrated Chl *a* autofluorescence, (**C**) PO_4_, (**D**) NO_3_, (**E**) total phytoplankton abundance (<20 µm), and (**F**) total phytoplankton biomass (<20 µm) in the upper water column of the North Atlantic during the STRATIPHYT II cruise. Black dots indicate individual sample depths, and gray circles represent the mixed layer depth. The figure was prepared using Ocean Data View (version 5.5.1). The white vertical dotted lines delineate the distinct regions of the cruise transect, characterized according to their physical and biochemical observations.

During the spring (April–May) of 2011, the upper water column of the study area ranged from weakly to non-stratified (based on $\bar{N^{2}}$ , Table S1) [30], with the latitudinal region between 29 and 48 °N characterized as weakly stratified with MLDs ranging from 11 to 59 (Figure 2). Higher latitudes stations (48–63 °N) were classified as well-mixed and were marked by deep MLDs ranging from 7 to 307 m (Table S1). Accordingly, seawater density and nutrient profiles demonstrated clear depth and latitudinal gradients (Figure 2A,D). Physicochemical characteristics and phytoplankton community dynamics have previously been discussed in detail [26,30] (Figure 2). Briefly, the ML of the southern region (29–40 °N) was characterized as oligotrophic based on nutrient concentrations (i.e., NO_3_ ≤ 0.13 µM and PO_4_ ≤ 0.03 µM). However, average Chl *a* concentrations (0.25 µg L^−1^), exceeded the <0.07 µg L^−1^ threshold previously used to define oligotrophic waters. Nutrient concentrations in the ML of the mid-latitude region (40–46 °N) remained low, averaging 0.07 ± 0.01 µM and 0.93 ± 0.21 µM for PO_4_ and NO_3_, with ML Chl *a* concentrations (averaging 1.16 ± 0.13 µg L^−1^) 4-fold higher compared to the southernmost region. In the northern well-mixed stations (48–63 °N), there was very little variability in nutrients and Chl *a* concentrations with depth, averaging 0.67 ± 0.02, 10.22 ± 0.32 µM, and 0.48 ± 0.03 µg L^−1^ for PO_4_, NO_3_, and Chl *a*, respectively.

Pico-sized (<20 µm) phytoplankton dominated PhytoA (comprising on average 97%) [30]. PhytoA was highest in the south and largely concentrated in the deep chlorophyll maximum (DCM), which shallowed with latitude, transitioning to a surface maximum in the mid-latitude regions (40–46 °N), which also corresponded with the peak in Chl *a* [30]. In the northern region, Phyto A and Chl *a* decreased corresponding to strong vertical mixing and deep mixing depths [30]. According to these physical (MLD, stratification level) and biochemical observations (i.e., NO_2_, NO_3_, PhytoA, PhytoC), the latitudinal transect was divided into 3 regions: Oligotrophic (29–40 °N), Bloom (40–46 °N), and Pre-bloom (48–63 °N). One exception was station 18 (48 °N); this station was classified as weakly stratified based on $\bar{N^{2}}$ and had a shallow MLD (7 m; compared to remaining well-mixed stations that ranged from 88 to 307 m), however, ML nutrient and Chl *a* concentrations were comparable to the northern well-mixed stations (Table S1) and thus, this station was classified as Pre-Bloom.

### 3.2. Heterotrophic Prokaryotes

#### 3.2.1. SURFACE

**Figure 3:**
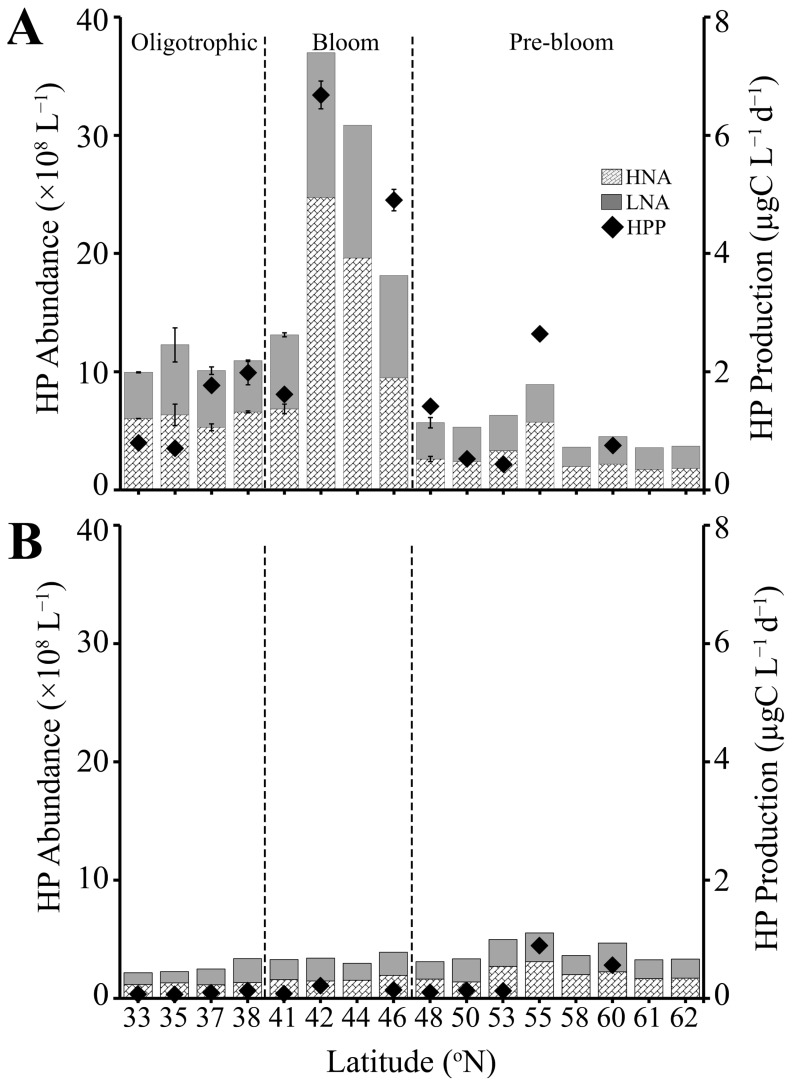
Heterotrophic prokaryote abundance of the HNA and LNA subpopulations (bar, ×10^8^ L^−1^) and total heterotrophic prokaryote production rate (dots, µgC L^−1^ d^−1^) measured by [^3^[^50^H]-leucine incorporation in (**A**) SURFACE (0–90 m) and (**B**) DEEP (90–200 m) samples. The dashed line separates three regions (Oligotrophic, Bloom, Pre-bloom). Error bar represents standard error.

Heterotrophic prokaryote abundance (HPA) displayed distinct latitudinal trends consistent with the above-described characteristics of the different regions (Table S2, Figure 3). SURFACE samples (0–90 m) from the Oligotrophic region had moderate HPA levels, averaging 9.8 ± 0.3 × 10^8^ L^−1^ (ranging from 5.0 to 14.6 × 10^8^ L^−1^),which increased nearly 1.6 fold in the Bloom region (average 15.4 ± 1.6 × 10^8^ L^−1^ ) and then decreased to the lowest values (average 5.2 ± 0.3 × 10^8^ L^−1^) in the deeply mixed Pre-bloom region. HP abundance and production were tightly coupled in SURFACE samples (Pearson’s correlation: N = 24, r = 0.90, *p*-value = 1.4 × 10^−9^). Accordingly, HPP was low in both the Oligotrophic and Pre-bloom regions, averaging 1.1 ± 0.2 and 1.0 ± 0.4 µgC L^−1^ d^−1^, respectively (Figure 3A), and increased 4-fold to an average of 3.9 ± 1.1 µgC L^−1^ d^−1^ in the Bloom region. Highest HPA (37 × 10^8^ cells L^−1^) and HPP (7.1 ± 0.06 µgC L^−1^ d^−1^) values were recorded in the Bloom region at station 13 (42 °N; Figure 3A). The average relative proportion of HNA and LNA cells (HNA:LNA) within SURFACE samples was 1.2 ± 0.04, 1.2 ± 0.05, and 1.1 ± 0.05 for the Oligotrophic, Bloom, and Pre-bloom regions, respectively.

#### 3.2.2. DEEP

In contrast to SURFACE samples, HPA in DEEP samples was relatively consistent with latitude and did not show clear trends across the different regions (Table S2, Figure 3B). Average HPA in DEEP samples was significantly reduced compared to SURFACE samples, i.e., 2.7, 4.9, and 1.4-fold for the Oligotrophic, Bloom, and Pre-bloom regions, respectively. HNA continued to marginally dominate the HP assemblage (HNA:LNA) of Oligotrophic (1.4 ± 0.1) and Pre-bloom regions (1.1 ± 0.03), with a slight dominance of LNA observed in DEEP samples of the Bloom region, where HNA:LNA decreased to an average 0.9 ± 0.04. Similar to HPA, HPP was consistently low in the DEEP samples. However, values were slightly elevated in the Pre-bloom region (0.36 ± 0.16 µgC L^−1^ d^−1^), compared to the Oligotrophic (0.20 ± 0.07) and Bloom (0.14 ± 0.04 µgC L^−1^ d^−1^) regions (Figure 3B). HPP maintained a significant correlation with HPA in the DEEP samples (Pearson’s correlation: N = 17, r = 0.70, *p*-value = 1.6 × 10^−3^).

### 3.3. Viruses

#### 3.3.1. SURFACE

**Figure 4:**
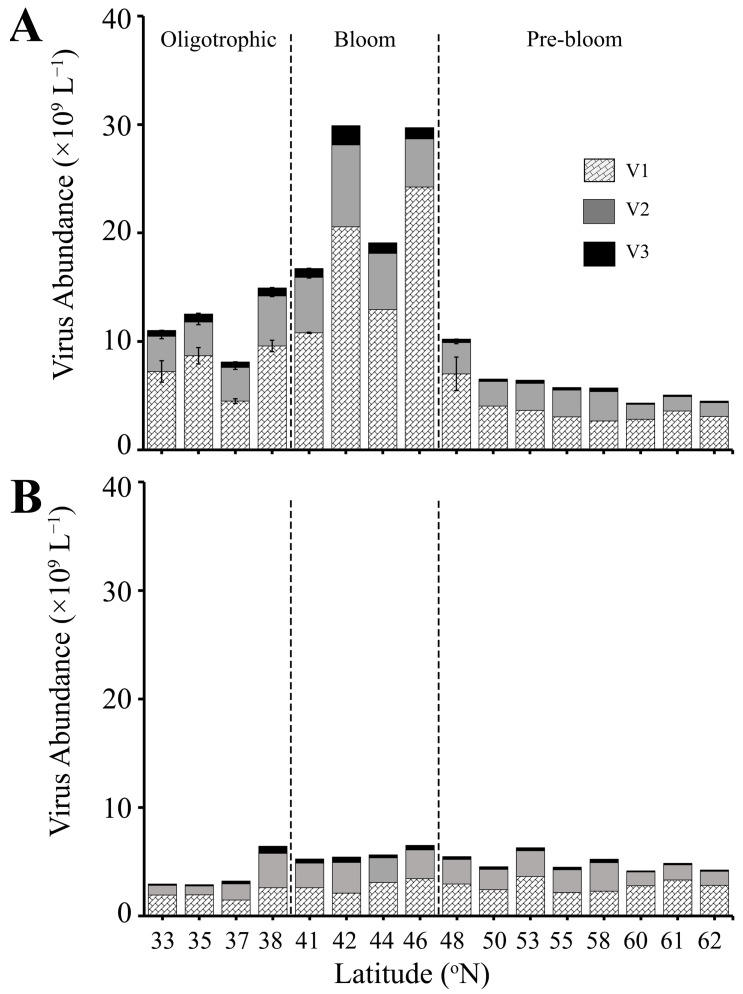
Abundance of virus subpopulations V1, V2, and V3 (×10^9^ L^−1^) at locations where viral production experiments were conducted. (**A**) SURFACE (0–90 m) and (**B**) DEEP (90–200 m) samples. The dashed line separates three regions (Oligotrophic, Bloom, Pre-bloom). Error bar represents standard error.

Total viral abundance (VA; i.e., sum of V1–V3) was highly correlated with HPA (Pearson’s correlation: N = 126, r = 0.83, *p*-value = 2.2 × 10^−16^) and HPP (Pearson’s correlation: N = 24, r = 0.83, *p*-value =4.7 × 10^−7^) in the SURFACE samples of all three regions (Table S3, Figure 4A). Specifically, VA in the Oligotrophic region was on average 10.5 ± 0.4 × 10^9^ viruses L^−1^, 14.2 ± 1.2 × 10^9^ L^−1^ in the mid-latitude Bloom region, and 6.9 ± 0.4 × 10^9^ viruses L^−1^ in the Pre-bloom region (Figure 4A). Similar to HPA and HPP, highest VA in surface samples was observed at station 13 (42 °N, 29.9 × 10^9^ L^−1^; Figure 4A). The numerically dominant V1 virus subpopulation comprised on average 60.8 ± 1.0%, 62.0 ± 1.5%, and 63.4 ± 1.4% of total virioplankton in the Oligotrophic, Bloom, and Pre-bloom regions, respectively. Overall (averaged across all 3 regions) V2 contributed on average 32.9 ± 0.7%, and V3 4.8 ± 0.2% of VA.

**Figure 5:**
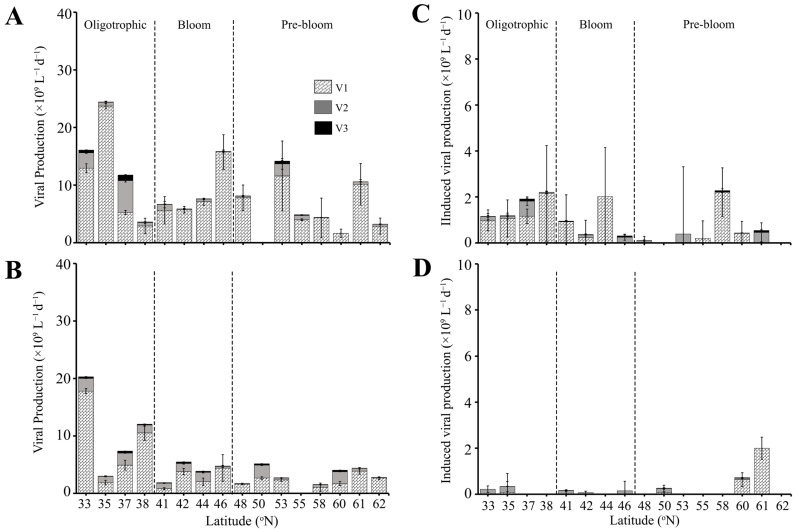
Lytic viral production rate (left panels) and the rate of induced viral production (right panels) for viral subpopulations V1, V2, and V3 in (**A**,**C**) SURFACE (0–90 m) and (**B**,**D**) DEEP (90–200 m) samples. The dashed line separates the three regions (Oligotrophic, Bloom, Pre-bloom). Note that panels (**C**,**D**) are on a different scale. Error bar represents standard error.

Total lytic virus production rate (VP) was highest in the Oligotrophic region, ranging from 2.9 to 41.1 × 10^9^ viruses L^−1^ d^−1^ (average 14.3 ± 5.6 × 10^9^ L^−1^ d^−1^) (Figure 5A, Table S3). VP in Bloom and Pre-bloom regions were comparable, with an average of 8.5 ± 1.9 × 10^9^ and 6.8 ± 1.6 × 10^9^ viruses L^−1^ d^−1^, respectively. Unlike VA, no correlation was observed for VP and HPA. Despite average V1 abundance being comparable across the 3 regions, the V1 subgroup had the lowest contribution to total VP in the Oligotrophic region (i.e., average 75.3 ± 8.9%, compared to 92.8 ± 4.1% and 92.4 ± 2.5% in the Bloom and Pre-bloom region, respectively; Figure S5A). Contributions of V2 to total VP were variable, ranging from 1.6% to 47.1% in the Oligotrophic region (average 20.7 ± 7.3%) and decreased to 6.7 ± 4.0% and 6.6 ± 2.2% in the Bloom and Pre-bloom regions, respectively (Table S3, Figure S5A). Contributions of V3 were on average between 0.6% and 4.0% across all three regions, with the highest contribution of 11.6% at 33 °N. Lytic production of V2 and V3 were negative at 60 °N (Table S3, not in figure).

Virus induction by Mitomycin C (i.e., VPI) was more variable than VP, i.e., not detected at all stations, with V2 having a higher occurrence of non-detectable VPI than V1. Total VPI was highest and most consistent in the Oligotrophic region (0.4–4.0 × 10^9^ L^−1^ d^−1^, average 1.6 ± 0.5 × 10^9^ viruses L^−1^ d^−1^) (Figure 5C), with virus induction on average 1.8- and 3.2-fold lower in the Bloom and Pre-bloom regions (Figure 5C, Table S3). VPI was dominated by V1 in the Oligotrophic and Bloom regions with an average contribution of 85.9 ± 5.7% and 64.7 ± 23.3%, respectively. V2 and V3 accounted for an average of 12.6 ± 5.1% and 1.6 ± 0.7% in the Oligotrophic and 28.4 ± 19.3% and 6.8 ± 4.1% in the Bloom region, respectively. Conversely, in the Pre-bloom region, V2 dominated over the V1, contributing 50.1 ± 17.9% to total VPI. VPI of V1 and V3 in this region was 42.0 ± 19.8% and 7.85 ± 3.14%. Overall viral production in the SURFACE layer was dominated by lytic production with contributions of 82.1 ± 7.1%, 88.6 ± 5.6%, and 90.8 ± 4.6% to the total viral production in the Oligotrophic, Bloom, and Pre-bloom regions (Figure S5C). This equates to around 44.7 ± 11.3% of the HP standing stock lysed by lytic infection, with lowest values for the Bloom region (22.2 ± 6.3%, compared to 57.0 ± 20.7% and 45.9 ± 21.1% for Oligotrophic and Pre-bloom regions, respectively). The only exception was at 38 °N, at the transition between Oligotrophic and Bloom regions where virus-induced production contributed 58% of the total viral production (Table S3). HP standing stock containing inducible lysogens was less variable between the regions, i.e., on average 7.4 ± 2.1%, 2.4 ± 1.4%, and 6.2 ± 3.7% in the Oligotrophic, Bloom and Pre-bloom regions, respectively.

#### 3.3.2. DEEP

Total VA in the DEEP samples was low with very little variation across the different regions (5.1 ± 0.5 × 10^9^, 7.1 ± 1.6 × 10^9^, and 5.5 ± 0.3 × 10^9^ L^−1^ in the Oligotrophic, Bloom, and Pre-bloom regions, respectively) (Figure 4B, Table S3). VA maintained a significant but weak correlation with HPA (Pearson’s correlation: N = 75, r = 0.35, *p*-value =2.2 × 10^−3^) and HPP (Pearson’s correlation: N = 17, r = 0.39, *p*-value =1.2 × 10^−1^). V1 contributions to total VA decreased in DEEP samples relative to the SURFACE, with an average contribution of 56.9 ± 2.2%, 55.8 ± 3.3%, and 60.6 ± 1.8% for the Oligotrophic, Bloom, and Pre-bloom regions, respectively. V2 contributions were highly variable, ranging from 8.0 to 58.9% of the total VA (average 36.4 ± 1.1%), and V3 contributions were consistently low, averaging 4.8 ± 0.2% across all the regions.

VP in DEEP samples of the Oligotrophic region was on average 1.3-fold lower (10.7 ± 3.7 × 10^9^ viruses produced L^−1^ d^−1^) than in the upper ocean (Figure 5B, Table S3). One notable exception was at 33 °N, where VP (20.3 × 10^8^ viruses L^−1^ d^−1^) was higher than the SURFACE sample at that same location (Figure 5A,B, Table S3). VP in DEEP samples within Bloom and Pre-bloom regions were slightly lower than SURFACE with 4.0 ± 0.8 × 10^9^ and 3.2 ± 0.5 × 10^9^ viruses L^−1^ d^−1^, respectively. V1 continued to dominate VP in the DEEP samples with average contributions of 76.0 ± 6.7%, 64.3 ± 10.8%, and 74.7 ± 7.9% in the Oligotrophic, Bloom, and Pre-bloom regions, respectively (Figure S5B). V2 contributions in DEEP samples were higher compared to SURFACE samples, with an average of 22% to 32% contributions to total VP. V3 contributions remained low, contributing on average 2–4% of the total VP (Figure S5B). Notably, V2 contributions were highest at 41 °N and 60 °N (51.6% and 51.1%, respectively), which were stations where V2 dominated VA. Similar to SURFACE samples, VP in the DEEP samples was not correlated to HPA and HPP.

In contrast to SURFACE, VPI in the DEEP samples was lowest in the Oligotrophic and Bloom regions, averaging 0.14 ± 0.08 × 10^9^ and 0.10 ± 0.04 × 10^9^ viruses L^−1^ d^−1^, respectively. Moreover, VPI was dominated by V2, which contributed on average 66.4 ± 14.3% of total VPI while V1 and V3 contributed 31.6 ± 14.2% and 1.9 ± 0.9%, respectively. VPI in Pre-bloom was comparable in DEEP and SURFACE samples, with average rates of 0.43 ± 0.28 × 10^9^ viruses L^−1^ d^−1^. 

Lytic production dominated total viral production in the DEEP layer (Figure S5D). Assuming a burst size 20, VP accounted in 209.5 ± 85.4%, 54.4 ± 14.2%, and 37.9 ± 9.7% of the HP standing stock being lysed per day in the Oligotrophic, Bloom, and Pre-bloom regions, respectively. The high standing stock lysed, exceeding total standing stock (i.e., 100%) in the Oligotrophic region, was largely driven by the relatively high VP at 33 °N (Figure 5B, Table S3). At this station, the percentage of standing stock lysed was 456.4%. However, removing this value, the standing stock lysed in the Oligotrophic region remained above 100%, with an average of 127.3 ± 32.5%. In comparison, on average, 3.1 ± 1.9%, 1.4 ± 0.6%, and 6.0 ± 4.2% of HP standing stock was infected with inducible lysogens in the Oligotrophic, Bloom, and Pre-bloom regions, respectively.

#### 3.3.3. VPI/VP

**Figure 6:**
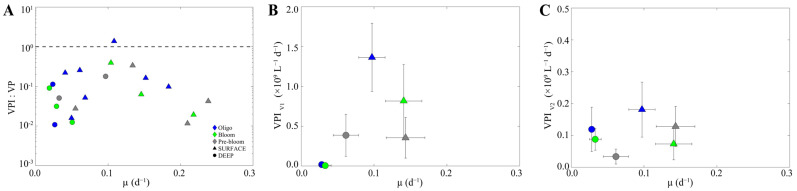
The relationship between the induced viral production (VPI) and heterotrophic prokaryote-specific growth rate (µ). (**A**) Variation in the relative proportion of lysogenic (VPI) to lytic viral production (VP) as a function of heterotrophic prokaryote-specific growth rate, shown on a logarithmic scale; the dashed line denotes VPI:VP = 1. (**B**) Induction rate of integrated V1 virus (VPI_V1_) and (**C**) V2 virus (VPI_V2_) subpopulations as a function of heterotrophic prokaryote-specific growth rate.

In order to assess changes in the relative proportion of lysogenic and lytic infection within the HP community with changes in host physiology, the ratio of VPI:VP was plotted against HP-specific growth rate for all stations and depths across the different regions. Interestingly, the relative proportion of VPI to VP increased with host-specific growth rate, reaching a tipping point at a growth rate of 0.1 d^−1^ when the rates were nearly equal; beyond this point, the VPI:VP ratio decreased with further increases in host growth (Figure 6A). This trend was largely driven by VPI, specifically changes in VPI_V1_ (Figure 6B,C). Moreover, when changes in host abundance and burst size, both of which vary positively with growth, were taken into account, the pattern persisted, supporting the conclusion that the trend was primarily driven by an increased proportion of lysogens within the HP community (Figure S6).

### 3.4. Ecosystem-Scale Connections

**Figure 7:**
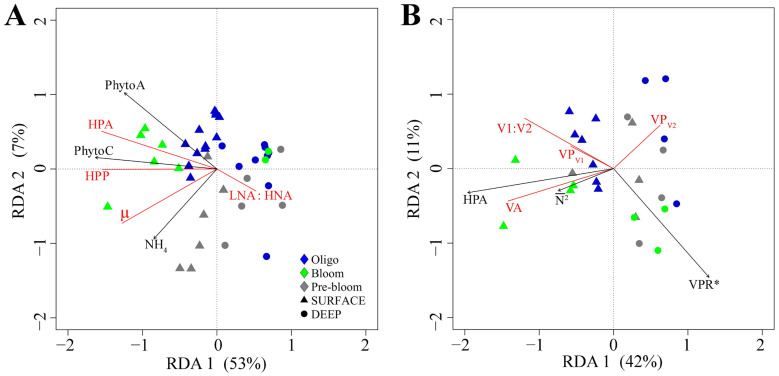
Redundancy analysis (RDA) correlation triplots of factors important in structuring the abundance and production of (**A**) heterotrophic prokaryotes and (**B**) viral subpopulations. Response variables are shown in red and explanatory variables that significantly (α = 0.1) contributed to the model in black. Symbols represent individual sampling points ((**A**): n = 40 and (**B**): n = 26), and color and shape are coded according to the specific region and depth level, respectively. The total variance explained by the RDA models were (**A**) 62% and (**B**) 58%, respectively.

A core interest of this study was to assess the hypothesis that changes in the physicochemical conditions that occur during the onset of the spring bloom impact HNA:LNA and HPP in a manner consistent with carbon availability. Figure 7A depicts the most parsimonious RDA model for H1. Forward selection revealed that PhytoA, PhytoC, NH_4_, and region significantly contributed to the model at α = 0.1 (Figure 7A, Table 1). The first two axes of the RDA triplot explained 53.4% and 7% of the variation in the data, with 62% of the total variability explained by the model. PhytoC (negative direction) was the main variable contributing to the formation of the first axis, while the second axis was driven by PhytoA (positive) and NH_4_ (negative). HPA, HPP, and µ were positively associated with stations with higher PhytoA and PhytoC and inversely correlated with DEEP samples with higher LNA:HNA. This suggests that variability in µ was an important factor regulating HPA and HPP. Moreover, it suggests that LNA may contribute less to HPP, particularly in more productive environments (Figure S2). DEEP samples are largely clustered together, whereas in SURFACE samples, ecosystem production increased progressively as regions shift from Pre-bloom to Oligotrophic to Bloom. Interestingly, SURFACE samples in the Pre-bloom region were associated with relatively high µ.

The RDA in Figure 7A indicates ecological and physiological differences in HP subpopulations, which may impact viral infection dynamics. Accordingly, RDA was used to evaluate the hypothesis (H2) that the abundance and VP of V1 and V2 depend on the relative abundance and production of HNA and LNA (Figure 7B, Tables S2 and S3). Forward selection revealed that HPA, subpopulation specific contact rate (VPR*), $\bar{N^{2}}$ , and region significantly contributed to the model at α = 0.1 (Figure 7B, Table 1). The first two axes in the RDA correlation triplot explained 42% and 11% of the total variation, with 58% of the total variation explained by the model. HPA (negative direction) was the main factor contributing to the formation of the first axis while the second axis was driven by $\bar{N^{2}}$ (negative) and VPR* (negative). In general, VA, *V*1:*V*2, and lytic production (VP) of V1 (VP_V1_) were positively associated with HPA and $\bar{N^{2}}$ . Additionally, VP_V1_ and *V*1:*V*2 were inversely related to VPR*. Conversely, lytic V2 (VP_v2_) was inversely related to VA, HPA, and $\bar{N^{2}}$ . These results suggest that host abundance and subpopulation specific contact rate between virus and host were important factors regulating the production and abundance of virus subgroups. Specifically, the abundance and production of V1 was associated with HNA hosts and environments with higher $\bar{N^{2}}$ . To further assess the link between virus and host subpopulations, linear regression analysis was conducted (Figures S3 and S4). Aligned with the RDA, the relative abundance (Figure S3) and production (Figure S4) of V1 was negatively correlated with LNA:HNA and positively related with the relative abundance of HNA cells. This link was most pronounced in samples from the Oligotrophic and Bloom regions.

## 4. Discussion

### 4.1. Heterotrophic Prokaryote Abundance and Production

The study area was characterized by dynamic physicochemical conditions consistent with the onset of seasonal stratification of the upper water column [58,59,60]. Accordingly, the meridional transect was divided into three distinct regions based on bio-physicochemical characteristics associated with the evolution of ecosystems during the annual phytoplankton bloom, which coincides with seasonal changes in stratification (for more details, see [30]). HPA, HPP, and µ were all tightly coupled to PhytoA and PhytoC. Due to the inherent link between the primary producers and HP consuming phytoplankton DOC (excretion, lysis, etc. [61,62]), it is not surprising that these variables co-vary across a wide range of aquatic ecosystems [63,64,65]. Moreover, since the bottom-up availability of DOC is considered as the primary factor regulating the activity of heterotrophic prokaryotes in much of the world’s oceans [66,67,68], the tight coupling between phytoplankton and heterotrophic prokaryotes suggests that food web processing of organic matter was driving the flux of available DOC. Specifically, the strong positive correlation between µ and NH_4_ (Figure 7A) suggests that grazing activity may be a key mechanism for enhancing the availability of DOC [69,70], particularly during the bloom period of the annual cycle [71].

HNA cells are generally considered the more active subgroup within HP communities and are often associated with higher cell-specific activity rates [20,24]. In line with this, our results reveal a positive correlation between the relative proportion of HNA cells in the community and both HPP and µ (Figure S2), suggesting that HNA cells exhibit higher metabolic activity [1,72,73]. Although community production assays and conversion factors, such as those based on leucine and thymidine incorporation, can vary across phyla [74] and may underrepresent LNA members [75,76], the observed increase in the relative abundance of HNA cells in tandem with ecosystem production (e.g., PhytoC, PhytoA, and HPA) provides additional support for this trend, independent of production measurements.

Phylogenetically, HNA cells are often closely related to copiotrophic members of the Bacteroidetes, Gammaproteobacteria, and Alphaproteobacteria [10,77,78]. Many of these groups are adept at responding to and utilizing proteins, peptides, and complex polysaccharides, particularly those linked to phytoplankton blooms [8,79,80,81,82]. This aligns with the higher HNA:LNA ratio observed in the Bloom region. Conversely, the relative proportion of LNA cells was positively associated with DEEP samples, as well as stations characterized by deep vertical mixing (Figure 7A). LNA cells are generally (though not always; [14]) associated with oligotrophic environments [83,84,85], a trend often attributed to the small, streamlined genomes of certain phyla within this subgroup (e.g., SAR11, [11,86]). These genomic features reduce the metabolic cost of replication and maintenance, conferring a competitive advantage under resource limitation [9,73,87,88]. LNA prokaryotes are also characterized by slower growth rates and a readily flexible surface-to-volume ratio, enabling them to maximize active surface area for efficient nutrient in low-nutrient environments [89]. Additionally, some LNA cells may possess specialized carbon storage mechanisms that allow them to accumulate carbon when available and conserve it when scarce [90], potentially conferring a competitive edge in deeper water layers and enabling a rapid response to episodic nutrient availability, such as that observed in blooming regions.

### 4.2. Virus Abundance, Producton, and Replication Mode

As viruses depend on their host to supply the energy and metabolic machinery needed for replication, their abundance and distribution closely align with that of their most prevalent hosts—heterotrophic prokaryotes [91]. Accordingly, we observe tight coupling between HPA and VA. Moreover, our results add to emerging evidence that ecological and physiological differences in HP subpopulations are key factors regulating viral infection dynamics [9,14]. Specifically, our study revealed that the abundance and production of V1 viruses increased with the potential encounter rates of HNA hosts (i.e., inverse VPR*), which was elevated in surface waters of more stratified stations of the Oligotrophic and Bloom regions. In contrast, the abundance and production of V2 viruses were positively associated with LNA cells, which were more prominent in DEEP samples and surface waters of stations characterized by deeper mixing (i.e., lower $\bar{N^{2}}$ ). This region-specific pattern suggests that seasonal and physical drivers, such as stratification and mixing, indirectly govern group-specific viral activity by modulating the distribution and ecological traits of host subpopulations.

Our data demonstrates that while lytic infection was the predominant mode of viral production within our study region, lysogeny was detected across all three regions of our study area. Moreover, the rate of induction of integrated viruses was found to increase with specific growth rate (µ) of HP until reaching a threshold of 0.1 d^−1^, after which it declined with further increases in growth. Although viral life-history traits (e.g., latent period and burst size) are influenced by resource availability and host physiology [92,93], the trend persisted even after normalizing VPI by HPA and accounting for burst-size variation with host growth (Figure S6). The positive trend at specific growth rates less than 0.1 d^−1^ is in opposition to previous studies that report that the frequency of inducible viruses was negatively correlated with host growth and abundance [94,95,96]. A similar trend was reported in a mesocosm study by Hu et al. (2025), wherein the initial increase in the frequency of lysogeny coincided with higher bacterial metabolic activity [97]. The fitness model of Li et al. (2020) suggests that lysogeny should be favored when susceptible host densities are low and the integrated virus confer direct fitness benefits to cellular growth and survival [98]. Accordingly, the positive relationship between host growth and the percentage of lysogens could be reflective of this fitness benefit conferred by temperate virus infection. Alternatively, under resource limitation of HP host, temperate phages often regulate lysogeny through the actions of phage-encoded repressors of the lytic cycle [99]. At growth rates greater than 0.1 d^−1^, intracellular ATP concentrations may be high enough for host proteases to clear phage repressors, favoring the lytic cycle [94,95].

To our knowledge, this is the first broad scale study to examine the potential influence of physiological and ecological differences between HNA and LNA cells on virus replication strategies, both in general and across different virus subpopulations. According to the traditional paradigm, one might expect lysogenic infection to be more prevalent in LNA-V2 host–virus systems (less productive host–virus system). Indeed, a high number of isolated Pelagiphages that infect SAR11 hosts are temperate [100,101,102]. Our data, however, reveals that changes in the rate of induced viral production were largely driven by the V1 virus subpopulation (Figure 6B,C), with no corresponding trend observed in V2. This again aligns with Hu and colleagues (2025), who reported that the increase in the frequency of lysogeny coincided with higher bacteria growth efficiency, suggesting that host bacteria were fast-growing and metabolically activity [97]. This suggests that lysogeny may be more important in r-selected host–virus pairs, contrary to the original paradigm that classified temperate phages as k-selected viruses [19]. R strategists are capable of rapid growth when resources are available, but abundances sharply decline when resources or environmental conditions change—thus exhibiting the characteristic boom and bust cycles. It would then follow that their viruses would benefit from mechanisms to cope with these fast changes in host availability and growth, whereas k-selective viruses (i.e., V2) follow a low and slow infection—tortoise strategy. Together, these results support the current hypotheses, which suggest that host genomic diversity, together with cellular metabolic state, governs the complex and often simultaneous occurrence of lytic and lysogenic infections in aquatic environment [97,103].

### 4.3. Organic Carbon Flow

Quantifying the contribution of viral lysis to carbon cycling is critical to understanding the fate of microbial biomass and the efficiency of the biological carbon pump. On average, 5.5 µgC L^−1^ of 11.7 µgC L^−1^ total available HP carbon, i.e., standing stock plus daily production, was lysed daily by lytic viral infection during our spring study. This corresponds to 47% of HP carbon being redirected to the DOM pool by viruses each day. This is consistent with prior estimates attributing 10–50% of surface ocean bacterial mortality to viral lysis [18,104,105,106]. Additionally, these values are ~10% higher than those observed during summer along the same transect [14], and more than 1.5-fold higher than spring and fall estimates for the Northwest Atlantic [9].

Comparing across regions, the highest values of carbon flux through the viral pathway were found in the Oligotrophic region, where 8.9 µgC L^−1^ of the 14.9 µgC L^−1^ of total available HP carbon was lysed in the surface waters, and 6.6 µgC L^−1^ of the 3.3 µgC L^−1^ in deep samples—equivalent to 59% and 202% of the available HP carbon pool, respectively. Conversely, the lowest values were observed in the Bloom region, where 5.4 µgC L^−1^ of 28.9 and 2.5 µgC L^−1^ of 4.5 µgC L^−1^ were lysed in surface and deep waters, respectively, corresponding to 19% and 55% of total available HP carbon lysed per day. These observations are consistent with prior reports of elevated turnover of HP biomass by lytic viral infection under stratified oligotrophic conditions in this region of the North Atlantic [14].

Values exceeding full turnover in the deep oligotrophic samples were driven by high viral lysis rates at 33 °N, which appeared decoupled from local host abundance and production. Such high lysis rates—exceeding the standing stock—are unlikely to result from in situ host production alone. This suggests that physical transport processes may have introduced infected cells into the deep layer, where lysis releases DOM directly into the mesopelagic. Several lines of evidence support this interpretation. Specifically, mixed layer depth, temperature, salinity, and nutrient concentrations in the deep layer were comparable to surface values at the same location (Table S1). Additionally, HNA:LNA ratios and relative contributions of virus subpopulations to lytic viral production (VP_*V*1:*V*2_) in these DEEP samples resembled those of SURFACE samples from the same location more than those of surrounding DEEP stations (Tables S2 and S3). This vertical injection of freshly lysed carbon may bypass the surface microbial loop, enhancing carbon export and the efficiency of the biological carbon pump by reducing rapid recycling near the surface.

**Figure 8:**
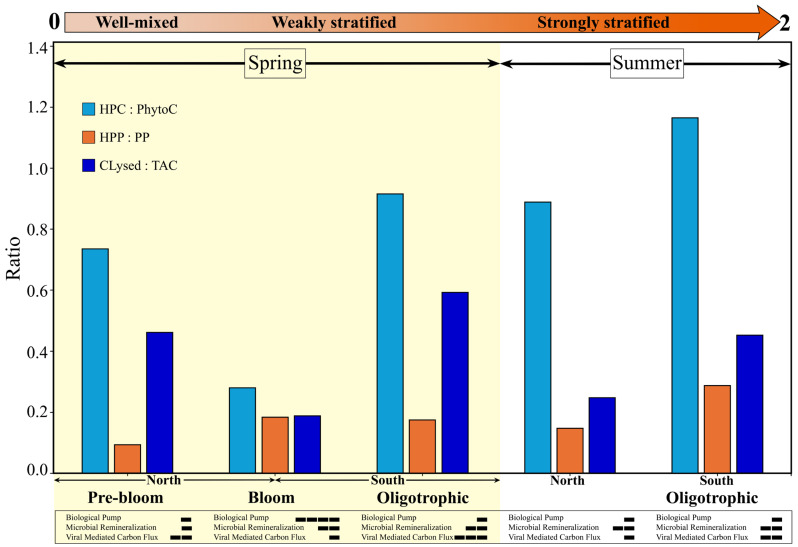
Data-derived conceptual overview of carbon flux through key biological pathways (i.e., biological carbon pump, microbial remineralization, and viral mediated carbon flux) in relation to seasonal shifts in vertical mixing and bio-physicochemical structure along a meridional transect of the North Atlantic Ocean. The figure highlights the dominant role of microbially mediated and virus-driven recycling of carbon under varying stratification regimes, with the highest potential for grazing and carbon export via the biological pump occurring during the spring bloom. Ratios represent the following: (1) the partitioning of carbon retained within heterotrophic prokaryote biomass (HPC; reflecting microbial recycling) relative to phytoplankton biomass (PhytoC; representing net autotrophic production); (2) the rate at which carbon is respired or recycled (heterotrophic prokaryote production, HPP) relative to that available for export through primary production (PP); (3) the fraction of heterotrophic prokaryote total available carbon (TAC; standing stock + daily production) that is lysed per day by lytic viral infection (Clysed). All data (Table S4) were obtained during the STRATIPHYT program and include heterotrophic production and loss during summer [14] and spring (this study; yellow shaded area), phytoplankton carbon [30], and primary production [26] across both seasons. The orange arrow depicts the transition in vertical stratification from well-mixed (0) to weakly stratified (1) and strongly stratified (2) conditions based on 100 m depth-averaged buoyancy frequency (N^2^) [30]. Horizontal bars represent the relative strength of the three microbial processes (outlined above) in regulating carbon flow across shifts in stratification.

Taken as a whole, the STRATIPHYT study highlights the dominant role of microbially mediated and virus-driven recycling of carbon, demonstrating how progressive changes in stratification and associated ecosystem structure regulate the balance between carbon recycling and export potential, with the greatest export capacity occurring under weakly stratified conditions (Figure 8, Table S4). Prior to the onset of stratification and bloom development, the biomass (HPC:PhytoC) ratio was estimated at 0.74, consistent with ratios (~0.7–1.0) commonly reported for open-ocean oligotrophic ecosystems [1,107]. This value indicates a moderately, microbially dominated system with limited capacity for grazing and carbon export through the biological carbon pump. It likely reflects the combined effects of residual deep mixing and light limitation constraining phytoplankton growth, coupled with elevated viral turnover of heterotrophic prokaryote carbon (as indicated by Clysed:TAC = 0.46). In agreement, the low production (HPP:PP) ratio (0.09) observed in this region reflects reduced autotrophic production under light-limited conditions, likely resulting in carbon limitation of heterotrophic prokaryotic production. At the onset of weak stratification, the biomass ratio drops to 0.28, indicating a dominance of new production and a large fraction of carbon in phytoplankton biomass (i.e., bloom development), leading to a higher potential for grazing and carbon export via the biological pump. As the nutrient limitation in the surface mixed layer of the oligotrophic regions constrains primary production, the ecosystem again moves towards a predominance of the microbial loop and viral-mediated recycling, as indicated by an average biomass ratio ~1.04 and fraction of total available carbon lysed (Clysed:TAC) ~0.52. Interestingly, the highest biomass (1.20) and production (0.29) ratios were observed in the southern oligotrophic region during the summer, likely sustained by elevated phytoplankton lysis reported in this region during the study. The enhanced release of dissolved organic carbon from virus-induced cell lysis would have fueled microbial remineralization, underscoring the strong viral regulation of phytoplankton biomass [71] and microbial carbon recycling. By integrating microbial stock and rate measurements of both production and loss, the STRATIPHYT study provides a clearer understanding of how microbial and viral interactions regulate the balance between carbon recycling and export across seasonal transitions.

## Figures and Tables

**Table 1:** Explanatory variables significant in the RDA correlation triplot of heterotrophic prokaryote (HNA, LNA) and viral subpopulations (V1, V2) in relation to environmental variables in Figure 7A,B. Significance was tested by a permutation test, using the multivariate pseudo-F (F) statistics using 9999 permutations [56].

Variable	AIC	Pseudo-F	*p*
HP Host subpopulation			
PhytoC	31.24	37.00	0.005
PhytoA	28.49	4.66	0.010
NH_4_	27.46	2.84	0.040
Region	27.32	1.86	0.100
Viral subpopulation			
HPA	27.74	12.66	0.005
VPR*	25.53	4.04	0.010
$\bar{N^{2}}$	24.77	2.47	0.050
Region	23.93	2.05	0.100

Abbreviations: AIC, Akaike Information Criterion; RDA, redundancy analysis.

## Data Availability

The original contributions presented in this study are included in the article and Supplementary Materials. Further inquiries can be directed to the corresponding authors.

## Supplementary Materials

The following supporting information can be downloaded at: https://www.mdpi.com/article/10.3390/microorganisms13112474/s1. Table S1. Physicochemical characteristics of stations where viral production experiments were conducted. Dashed lines separate regions. (*Abbreviation*—*Lat*, Latitude; *MLD*, mixed layer depth; *Strat Level*, water column stratification level, non-stratified, 0, and weakly stratified, 1; *Temp*, temperature; *K_T_*, turbulence; $\bar{N^{2}}$ , 100 m depth-averaged buoyancy frequency, Chl *a*, Chlorophyll *a*). Table S2: Heterotrophic prokaryote (HP) subpopulations abundance (HNA, LNA, ×10^8^ L^−1^), relative ratio of HNA and LNA subpopulation (HNA:LNA), total community production (HPP, µgC L^−1^ d^−1^), and specific growth rate (µ, d^−1^). Dashed lines separate regions. Table S3. Lytic- and mitomycin C-induced viral production data of virus V1, V2, and V3 subpopulations. (*Abbreviation*—*Lat*, latitude; *V*1:*V*2, relative ratio of V1 and V2 virus population abundance; *VPR**, subpopulation-specific virus to prokaryote ratio; *VP*, viral production rates due to lytic infection; *VPI*, mitomycin C-induced lysogenic viral production). Dashed lines separate regions. Table S4: Average carbon biomass and daily carbon flux (both in units of µg C L^−1^) of key microbial components observed during spring (Pre-bloom: 48–63 °N; Bloom: 40–46 °N; Oligotrophic: 29–40 °N) and summer (North: 30–45 °N; South: 45–63 °N) along the meridional transect of the North Atlantic. These values were used in the ratio calculations presented in Figure 8. All data were obtained during the STRATIPHYT program. Note: Reference numbers in the Supplementary Information follow the numbering used in the main manuscript. Abbreviations: *PhytoC*, phytoplankton carbon biomass; *PP*, primary production; *HPC*, heterotrophic prokaryote biomass; *HPP*, heterotrophic prokaryote production; *VC*, viral biomass; *TAC*, total available carbon (HPC + HPP); *Clysed (lytic)*, carbon lysed from lytic infection; *Clysed (lysogenic)*, carbon lysed from lysogenic infection. Figure S1. Representative flow cytograms of heterotrophic prokaryote (A) and viral (B) subpopulations in seawater, distinguished by green fluorescence (530 nm) and right-angle light scatter (488 nm) following staining with SYBR Green I. Figure S2. Heterotrophic prokaryotes subpopulation (HNA and LNA) abundance and production regression analysis. Mean regression of (A) HP abundance versus relative proportion of HNA and LNA cells, (B) the ratio of HNA and LNA cells versus HP production, (C) HP production per cell or specific growth rate per day (µ) as a function of the relative proportion of HNA to LNA cells. Error bar represents standard error. Figure S3. Link between the relative abundance of HNA and LNA cells and the relative abundance of V1 and V2 virus subpopulations. Figures illustrate group- and activity-specific linkages between host and virus subpopulations, specifically (A) V2-LNA and (B) V1-HNA. Error bar represents standard error. Figure S4. Link between the relative abundance of HNA and LNA cells and the relative contribution of V1 and V2 to total lytic production rates. Figures illustrate group- and activity-specific linkages between host and virus subpopulations, specifically (A) V2-LNA and (B) V1-HNA. Error bar represents standard error. Figure S5. Relative contribution of the three viral subpopulations to total lytic production, and the relative contribution of lytic and lysogenic infections to total viral production rates in surface (0–90 m; panels A and C) and deep (90–200 m; panels B and D) samples. Dashed lines indicate the boundaries of the three regions (Oligotrophic, Bloom, Pre-bloom). Figure S6. Percentage of lysogens calculated using a host growth rate-dependent burst size. For heterotrophic prokaryote-specific growth rates (µ) < 0.1 d^−1^, a burst size of 13 was applied; for µ ≥ 0.1 d^−1^, a burst size of 20 was used.
