# Supplementary material for: Heterotrophic Prokaryote Host–Virus Dynamics During Spring in the Northeast Atlantic Ocean
Source: Microorganisms. 2025 Oct 29;13(11):2474. doi: 10.3390/microorganisms13112474 (PMC12654298; doi:10.3390/microorganisms13112474)

Heterotrophic Prokaryote Host-Virus Dynamics During Spring in the Northeast Atlantic Ocean

Yean Das ^1^, Corina P. D. Brussaard ^2,3^ and Kristina D. A. Mojica ^1,^ *

**Table S1.** Physicochemical characteristics of stations where viral production experiments were conducted. Dashed lines separate regions. (*abbreviation*- *Lat*, Latitude; *MLD*, mixed layer depth; *Strat* *Level*, water column stratification level, non-stratified, 0 and weakly stratified, 1; *Temp*, temperature; *K_T_*, turbulence; $\overline{N^{2}}$, 100m depth averaged buoyancy frequency, *Chl* *a*, Chlorophyll a).

**
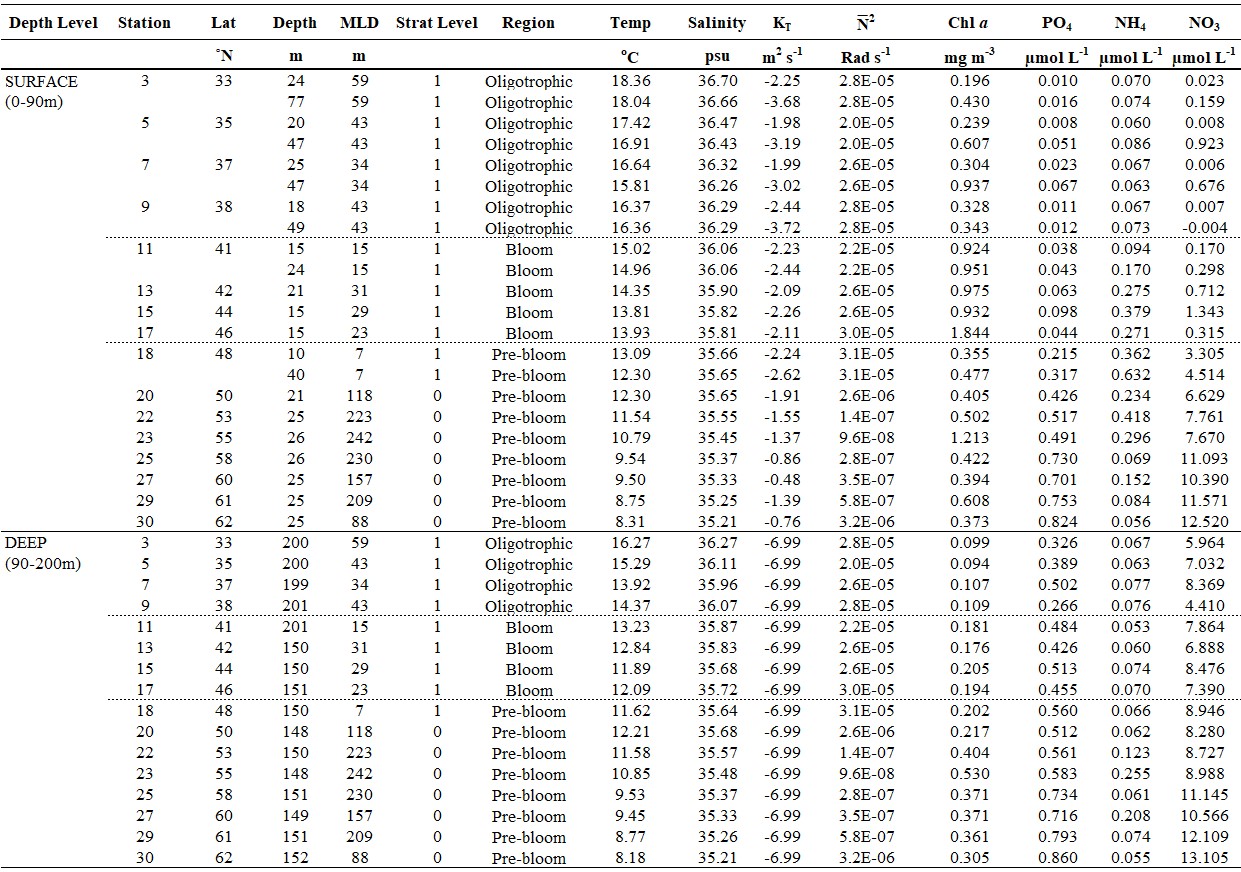
**

**Table S2**: Heterotrophic prokaryote (HP) subpopulations abundance (HNA, LNA, x10^8^ L^-1^), relative ratio of HNA and LNA subpopulation (HNA:LNA), total community production (HPP, µgC L^-1^ d^-1^) and specific growth rate (µ, d^-1^). Dashed lines separate regions.


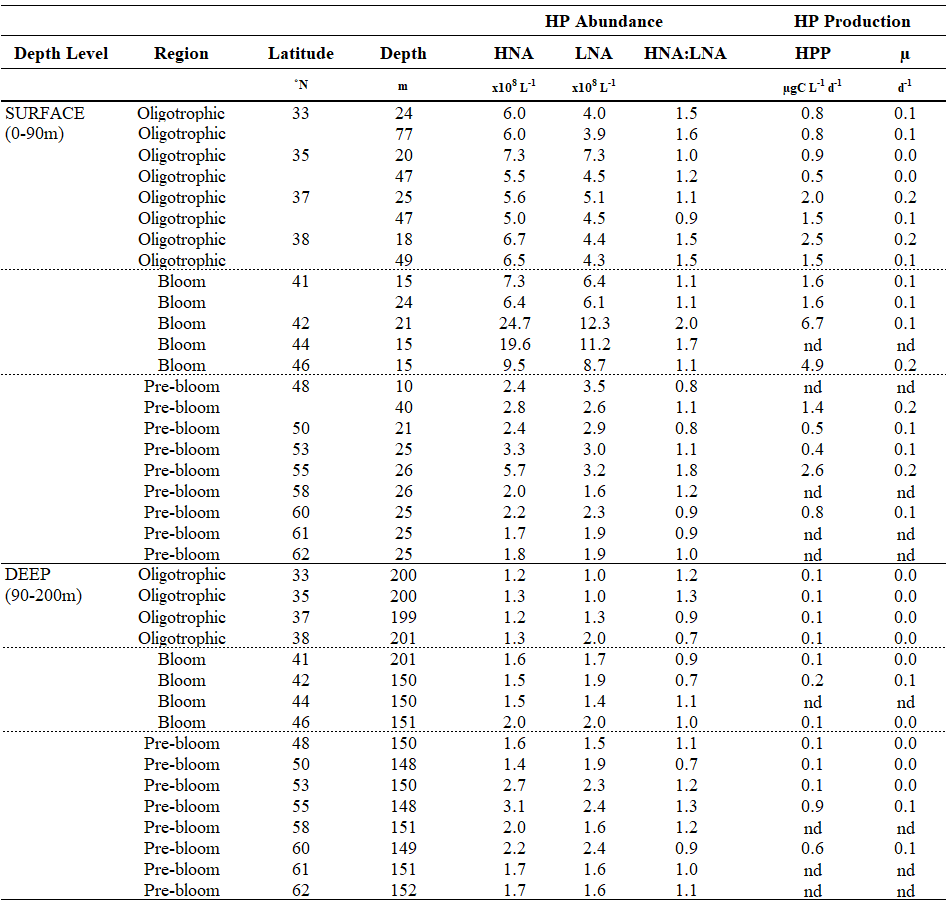


**Table S3.** Lytic and mitomycin C induced viral production data of virus V1, V2 and V3 subpopulation. (*abbreviation*- *Lat*, latitude; *V1:V2*, relative ratio of V1 and V2 virus population abundance; *VPR*,* subpopulation specific virus to prokaryote ratio; *VP*, Viral production rates due to lytic infection; *VPI*, mitomycin C induced lysogenic viral production). Dashed lines separate regions.


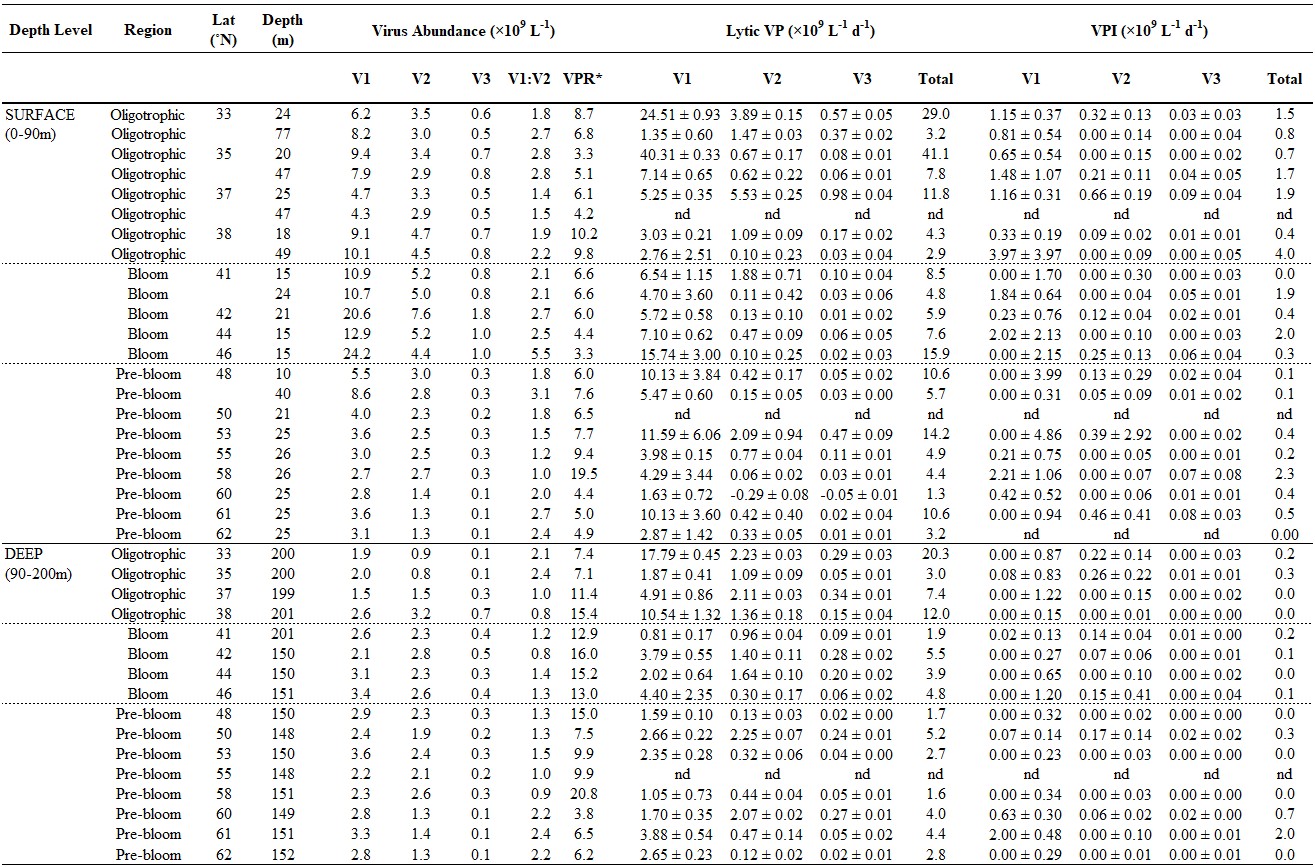


**Table S4:** Average carbon biomass and daily carbon flux (both in units of µgC L⁻¹) of key microbial components observed during spring (Pre-bloom: 48–63° N; Bloom: 40–46° N; Oligotrophic: 29–40° N) and summer (North: 30–45° N; South: 45–63° N) along the meridional transect of the North Atlantic. These values were used in the ratio calculations presented in Figure 8. All data were obtained during the STRATIPHYT program. Note: Reference numbers in the Supplementary Information follow the numbering used in the main manuscript. Abbreviations: *PhytoC*, phytoplankton carbon biomass; *PP*, primary production; *HPC*, heterotrophic prokaryote biomass; *HPP*, heterotrophic prokaryote production; *VC*, viral biomass; *TAC*, total available car-bon (HPC + HPP); *Clysed (lytic)*, carbon lysed from lytic infection; *Clysed (lysogenic)*, carbon lysed from lysogenic infection.

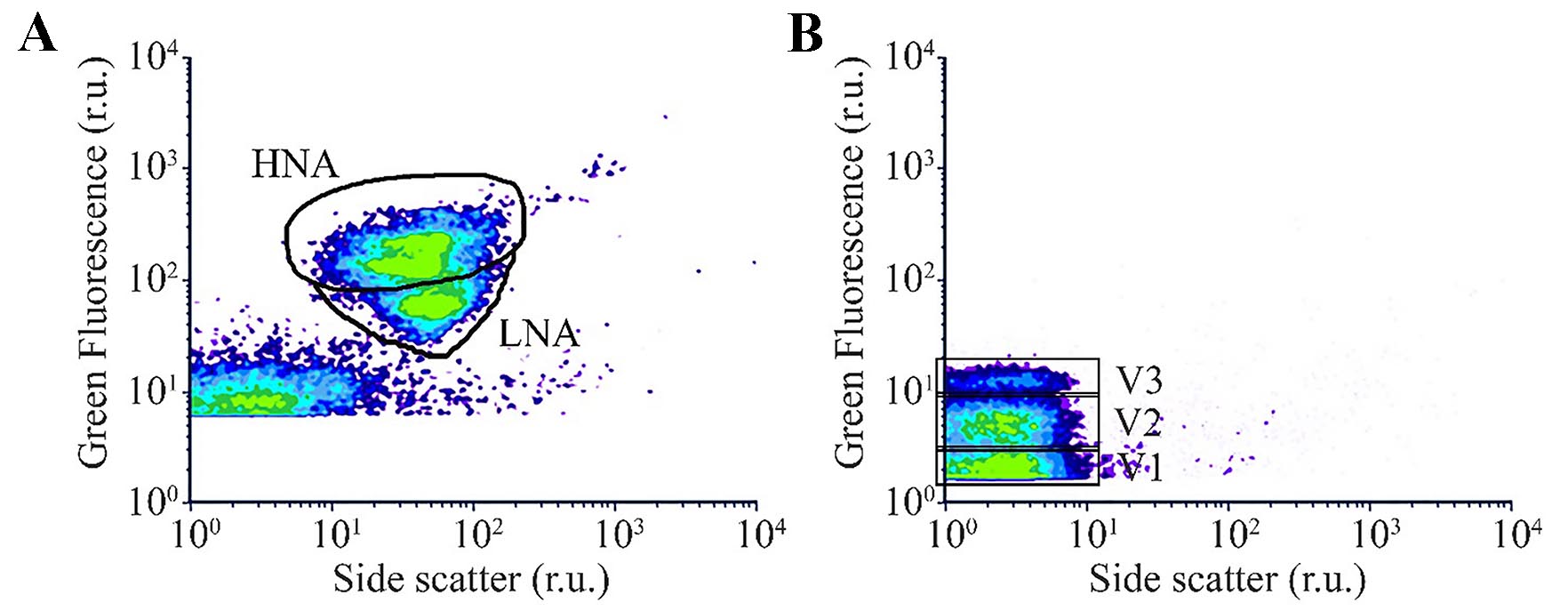
**Figure S1:** Representative flow cytograms of heterotrophic prokaryote (**A**) and viral (**B**) subpopulations in seawater, distinguished by green fluorescence (530 nm) and right-angle light scatter (488 nm) following staining with SYBR Green I.

**Figure S2:** Heterotrophic prokaryotes subpopulation (HNA and LNA) abundance and production regression analysis. Mean regression of (**A**) HP abundance versus relative proportion of HNA and LNA cells, (**B**) the ratio of HNA and LNA cells versus HP production, (**C**) HP production per cell or specific growth rate per day (µ) as a function of the relative proportion of HNA to LNA cells. Error bar represents standard error.


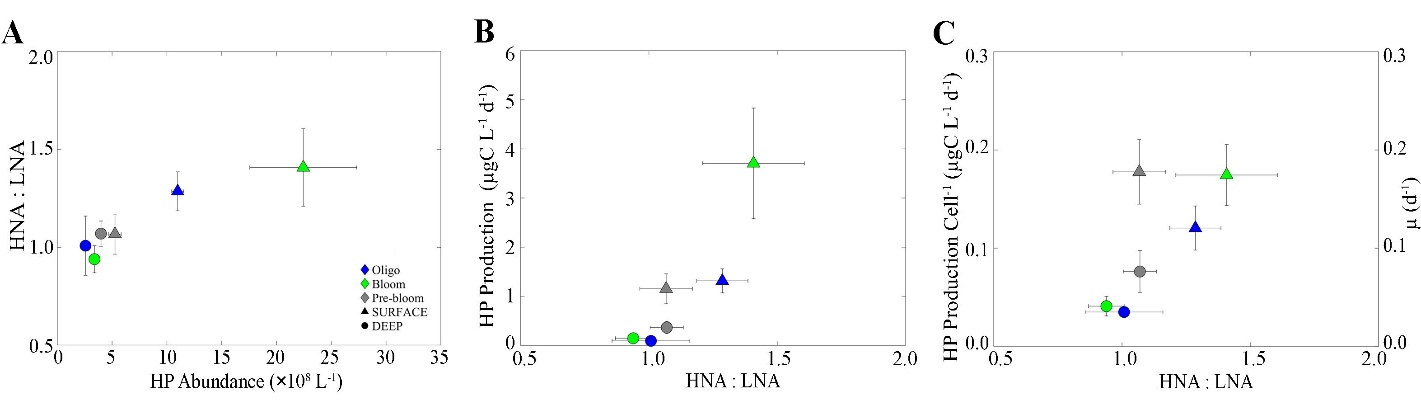


**Figure S3:** Link between the relative abundance of HNA and LNA cells and the relative abundance of V1 and V2 virus subpopulations. Figures illustrate group- and activity-specific linkages between host and virus subpopulations specifically (**A**) V2-LNA and (**B**) V1-HNA. Error bar represents standard error.


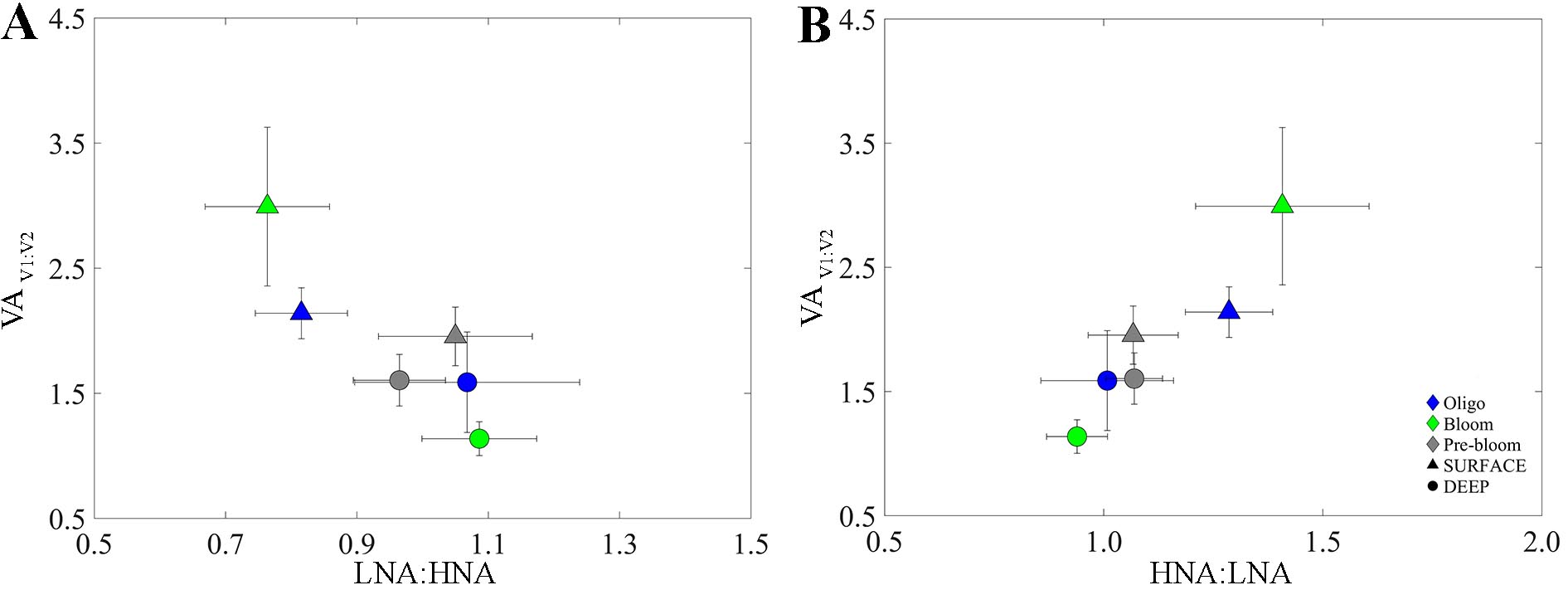


**Figure S4:** Link between the relative abundance of HNA and LNA cells and the relative contribution of V1 and V2 to total lytic production rates. Figures illustrate group- and activity-specific linkages between host and virus subpopulations specifically (**A**) V2-LNA and (**B**) V1-HNA. Error bar represents standard error.


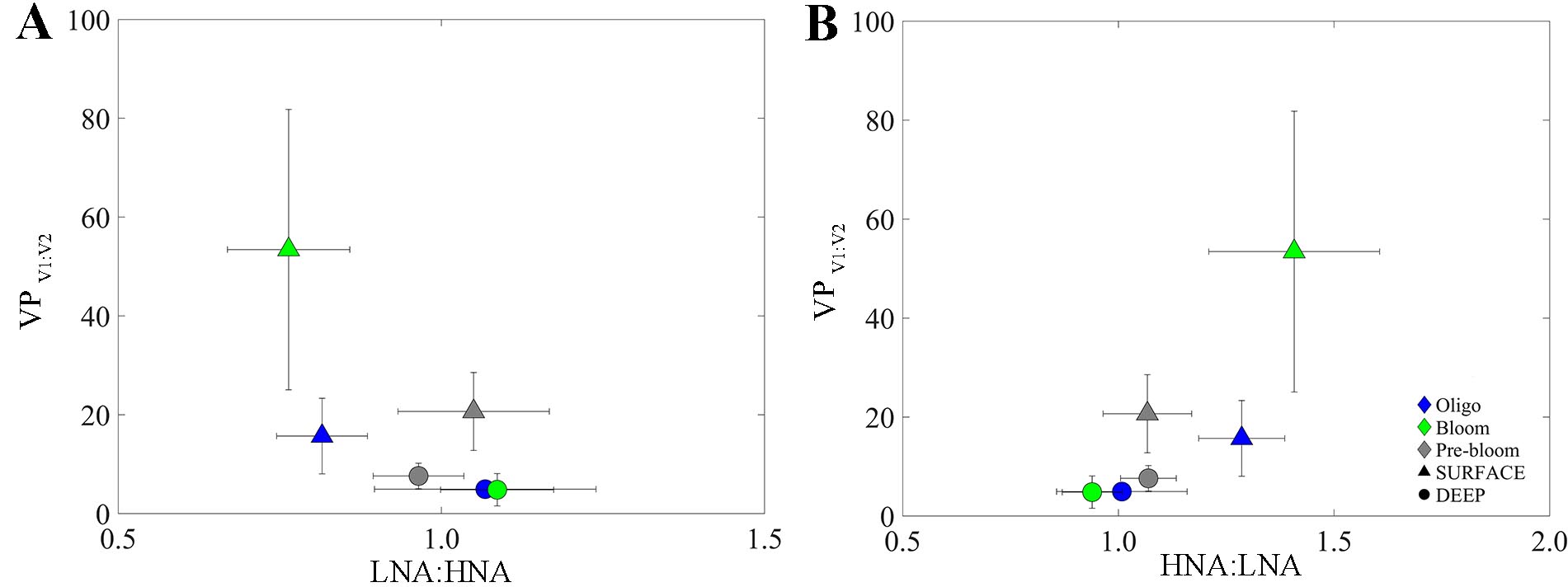


**Figure S5:** Relative contribution of the three viral subpopulations to total lytic production, and the relative contribution of lytic and lysogenic infections to total viral production rates in surface (0–90 m; panels **A** and **C**) and deep (90–200 m; panels **B** and **D**) samples. Dashed lines indicate the boundaries of the three regions (Oligotrophic, Bloom, Pre-bloom).


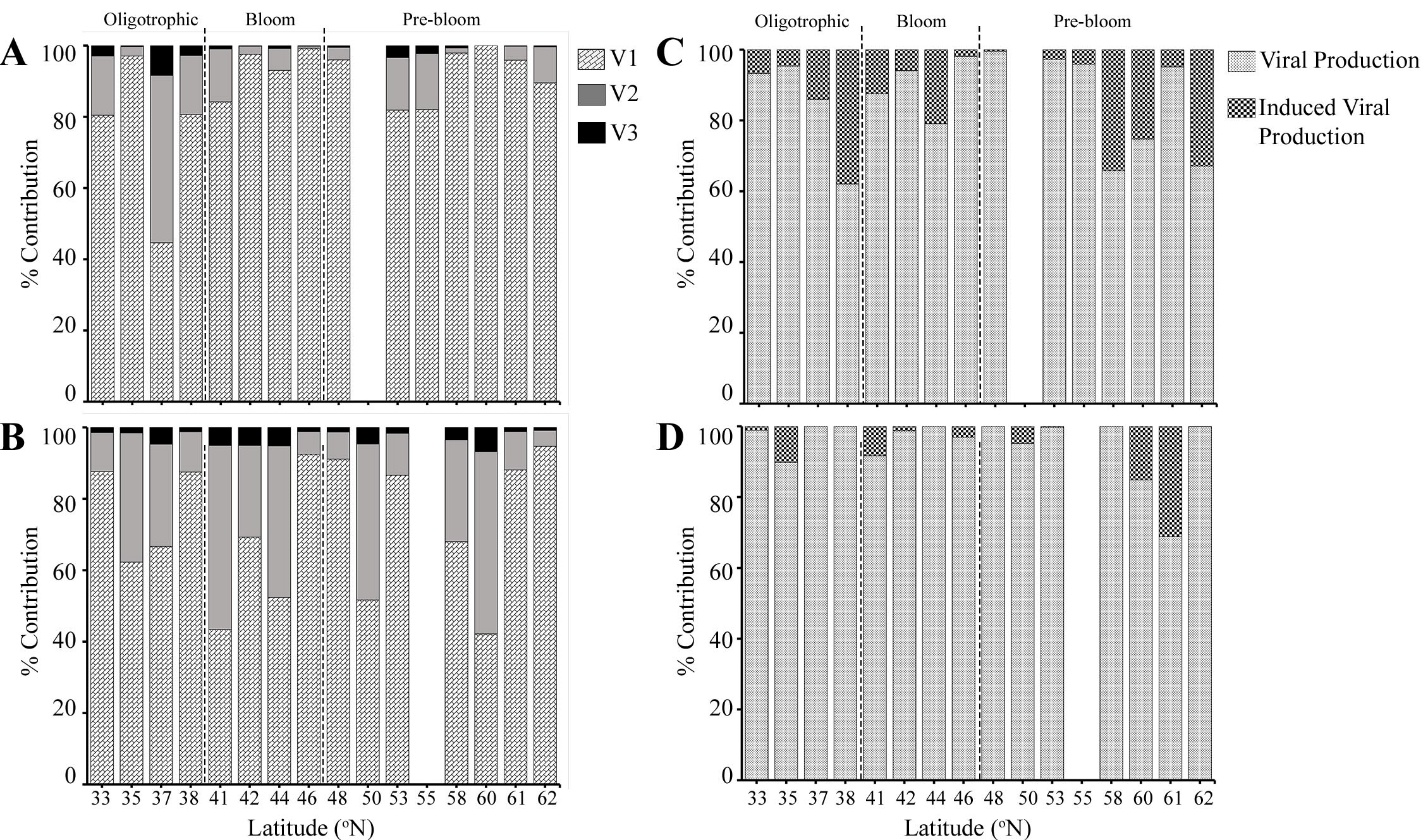


**Figure S6:** Percentage of lysogens calculated using a host growth rate–dependent burst size. For heterotrophic prokaryote specific growth rates (µ) < 0.1 d⁻¹, a burst size of 13 was applied; for µ ≥ 0.1 d⁻¹, a burst size of 20 was used.


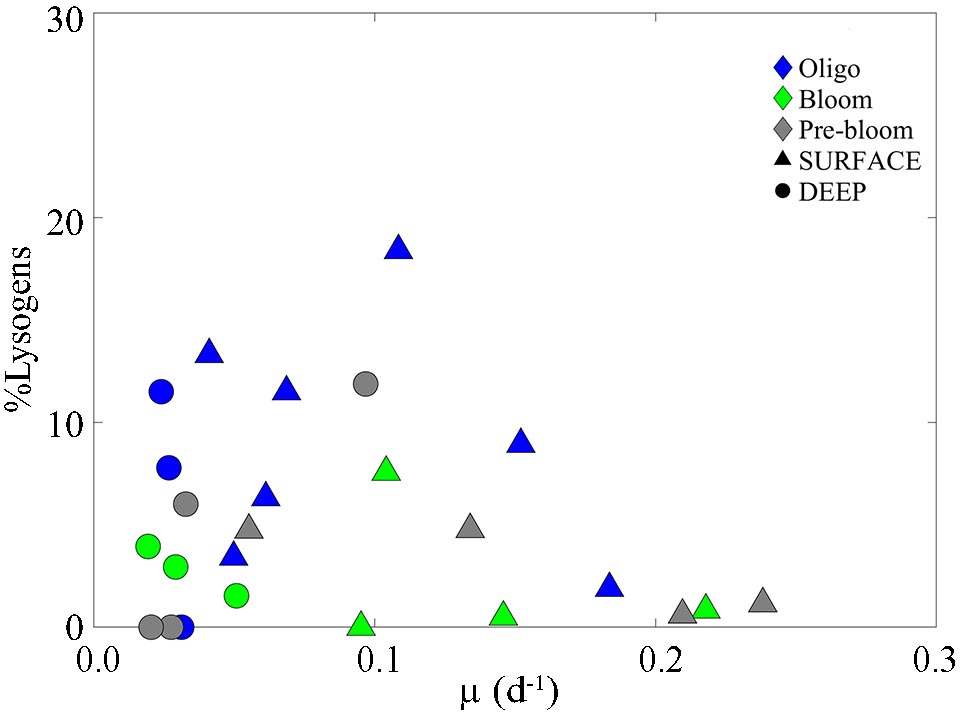

Supplement: Supplementary file 1 [file microorganisms-13-02474-s001.zip › microorganisms-3874403_Supplementary_proofreading/microorganisms-3874403_supplement_revised_FINAL.docx]
