# Supplementary figures and images for: Heterotrophic Prokaryote Host–Virus Dynamics During Spring in the Northeast Atlantic Ocean
Source: Microorganisms. 2025 Oct 29;13(11):2474. doi: 10.3390/microorganisms13112474 (PMC12654298; doi:10.3390/microorganisms13112474)

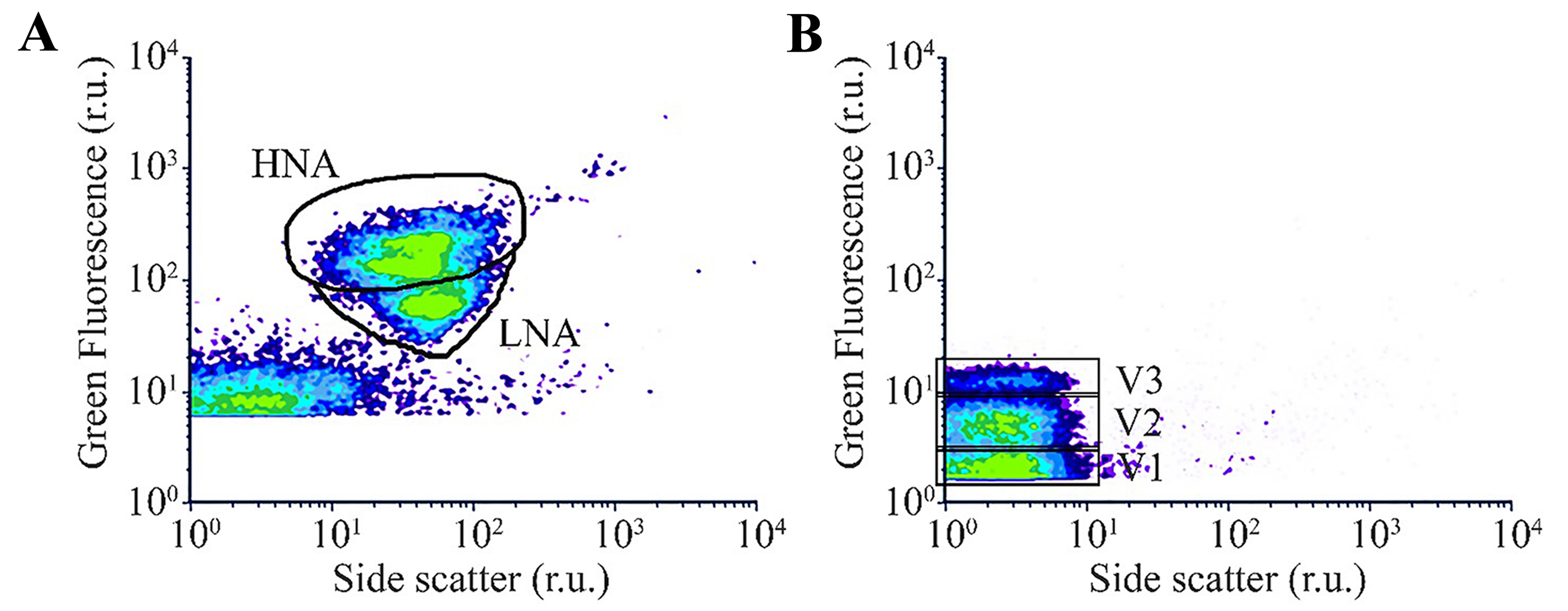

Supplement: Supplementary file 1 [file microorganisms-13-02474-s001.zip › microorganisms-3874403_Supplementary_proofreading/S1. Cytograph Plot.jpg]

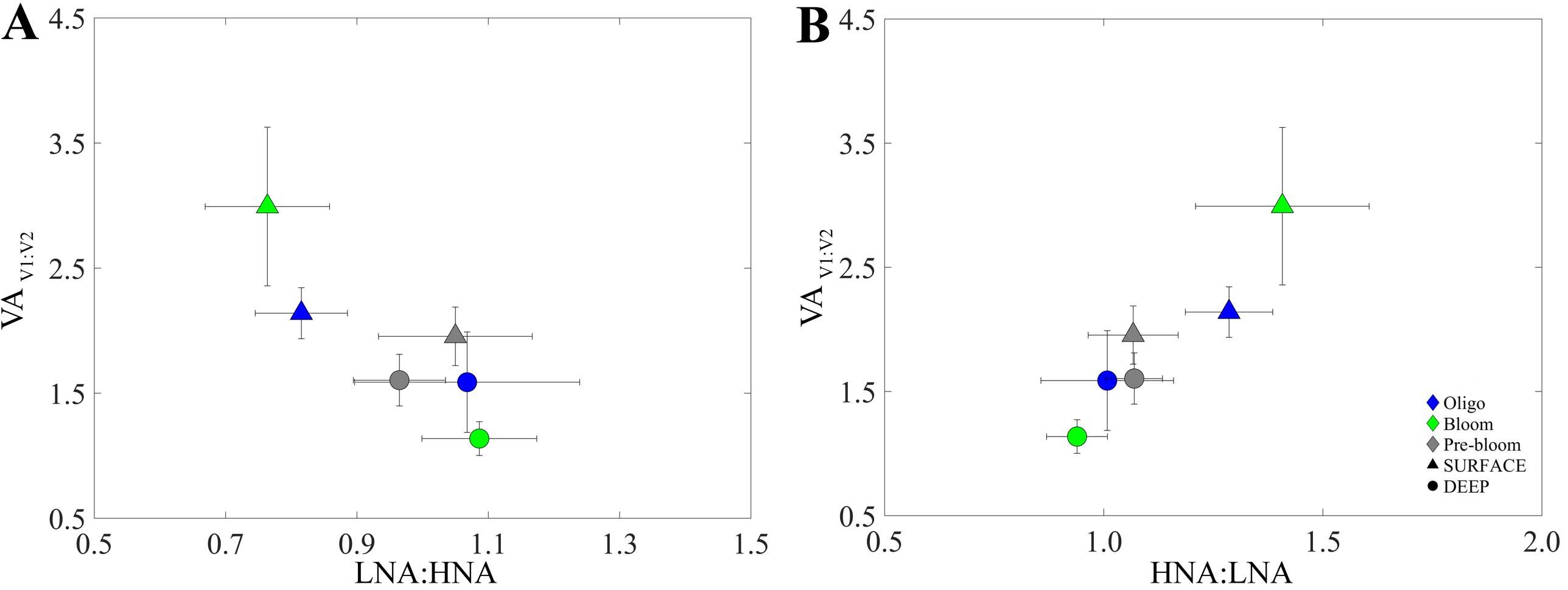

Supplement: Supplementary file 1 [file microorganisms-13-02474-s001.zip › microorganisms-3874403_Supplementary_proofreading/S3. HNALNA-LNAHNA-VA1VA2.jpg]

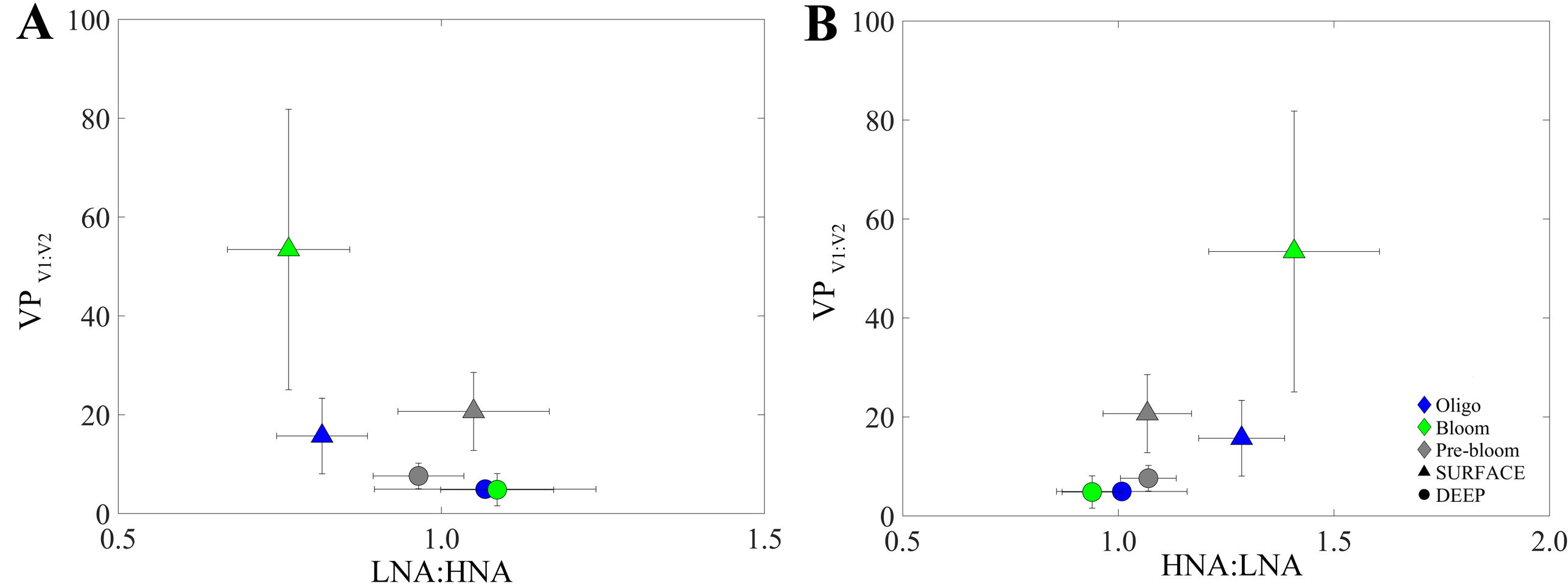

Supplement: Supplementary file 1 [file microorganisms-13-02474-s001.zip › microorganisms-3874403_Supplementary_proofreading/S4. HNALNA-LNAHNA-VP1VP2.jpg]

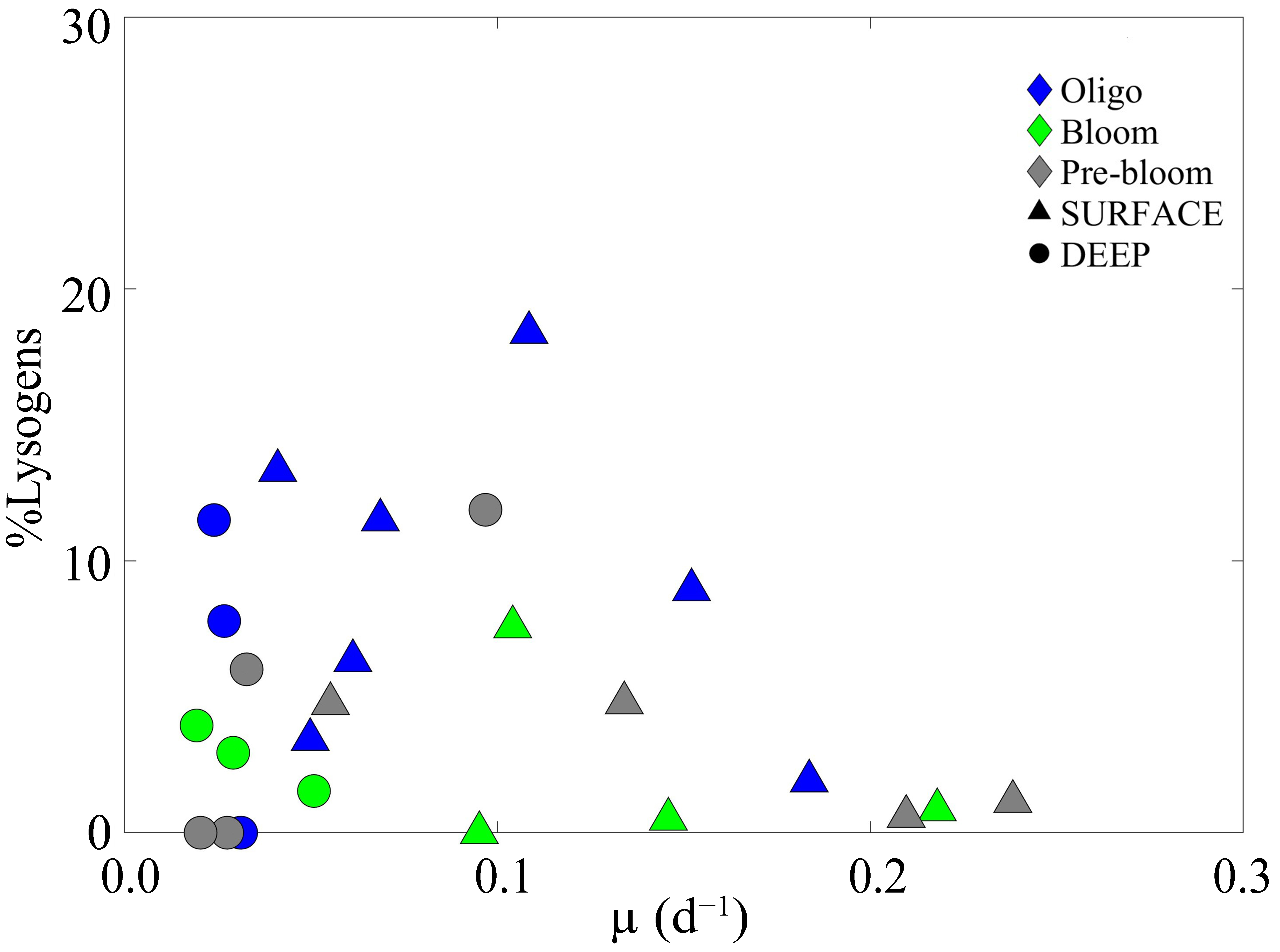

Supplement: Supplementary file 1 [file microorganisms-13-02474-s001.zip › microorganisms-3874403_Supplementary_proofreading/S6. %Lysogens with host growth.jpg]
